# Seasonal progression of melt and snowlines in Alaska from SAR reveals impacts of warming

**DOI:** 10.1038/s41612-026-01321-y

**Published:** 2026-02-04

**Authors:** Albin Wells, David R. Rounce, Mark Fahnestock

**Affiliations:** 1 Department of Civil and Environmental Engineering, Carnegie Mellon University, Pittsburgh, PA USA; 2 Geophysical Institute, University of Alaska Fairbanks, Fairbanks, AK USA

**Keywords:** Climate sciences, Environmental sciences, Natural hazards

## Abstract

Glaciers in Alaska contribute greatly to sea-level rise and are losing mass at a faster rate than any other region. Yet, our understanding of ongoing changes and ability to model them are hindered by a lack of observations, particularly at high spatiotemporal resolution. Here, we leverage Sentinel-1 synthetic aperture radar (SAR) data to produce temporally-varying glacier melt extents and snowlines from mid-2016 to 2024 for 99% of glaciers in Alaska greater than 2 km^2^. The melt extents are strongly correlated with temperatures, revealing that each 1°C increase in summer temperature causes up to 3 additional weeks of glacier melt. The high spatiotemporal resolution also captures subseasonal changes such as the 2019 heat wave, which caused subregional snowlines to retreat up to 105 m higher and exposed up to 28% more of the underlying glacier compared to typical years. Our snowlines agree well with optical datasets (r^2^ up to 0.94), thus providing unprecedented reliable data unencumbered by clouds or lighting conditions. Moving forward, our automated, open-source workflow can easily be applied to other regions. These data also present unique opportunities to calibrate and validate large-scale glacier evolution models, a critical step for improving projections of glacier changes and their impacts.

## Introduction

Mountain glaciers contribute greatly to sea-level rise, are an important water resource, and act as crucial sentinels of climate change^1–3^. In Alaska, glaciers have lost more mass from 2000 to 2023^3,4^ and are projected to contribute more to sea-level rise by 2100^5^ than any other mountain glacier region. Accurate observations of these changes are necessary to understand the drivers of glacier mass loss and improve projections, especially in light of the rapidly changing Arctic climate^6^ and extreme events, such as heat waves, that have a substantial impact on mountain glaciers^7–9^.

Satellite-based observations are a powerful tool for monitoring glacier changes globally^10^, especially those that provide insight into glacier mass change^3^. Transient and seasonal snowlines can estimate the glacier equilibrium-line altitude, i.e., the highest elevation on the glacier where the climatic mass balance is zero for a given year^11^, which is closely related to annual mass balance^12–15^. Mapping changes in transient snowlines and equilibrium-line altitudes over time thus elucidates drivers of glacier change^16–25^ and improves glacier models’ abilities to predict interannual and subannual changes^26–29^.

Currently, all large-scale systematic efforts to derive snowlines utilize optical data^22,25,30–33^, which suffer from inconsistent sampling due to cloud cover, light, and snow events. These challenges are often exacerbated on mountain glaciers in late summer, which is particularly problematic considering the importance of capturing the maximum snowline to properly estimate equilibrium-line altitudes^24,34,35^. Furthermore, fully-automated workflows to obtain glacier snowlines from optical data often suffer from large errors due to optical similarities between snow and clouds, as well as cloud shadows and shading effects that complicate the classification of debris, ice, firn, and snow^30^.

Synthetic aperture radar (SAR) is a robust and reliable remote sensing observation since it penetrates clouds and functions regardless of light. The strength of the SAR signal return, known as backscatter, depends entirely on the physical properties of the surface. On glaciers in the winter, SAR penetrates dry snow and scatters off the underlying ice or debris in the ablation zone, and ice lenses in the firn of accumulation areas. During the summer, water in the snow pack absorbs radar wave energy, which reduces backscatter and reveals the dry-to-wet snowline (i.e., melt extent)^36,37^. As the snowline retreats, the underlying ice or firn is exposed and backscatter increases, providing an estimate of the snowline. Existing studies have demonstrated the potential for SAR to derive temporally-varying melt extents^38–45^ and snowlines^46–51^; however, studies have yet to systematically process continuous snowline time series from SAR at regional scales. Ultimately, these data offer invaluable insights into the onset, duration, and severity of the ablation season, and serve as important calibration and/or validation data for temperature reanalyses and glacier models.

In this study, we use Sentinel-1 SAR data to map transient snowlines and glacier melt extents for 99% of glaciers in Alaska greater than 2 km^2^, representing 85% of the total glaciated area in Alaska. These data capture detailed spatial and temporal variations in melting and snowline retreat across the region. We showcase the power of these data by quantifying the impact of the 2019 heat wave in Alaska on snowline retreat, underscoring the sensitivity of glaciers to short-term climatic variability. Our observations show strong agreement with snowlines derived from optical sources (r^2^ up to 0.94) and are remarkably robust, as SAR-based observations are unaffected by cloud cover and lighting conditions. These observations provide an unprecedented basis for high temporal-resolution data developed via an automated workflow that can be applied to other regions, and improve our ability to model large-scale glacier changes on subseasonal scales.

## Results

### Patterns of glacier melt across and within subregions

**Fig. 1:**
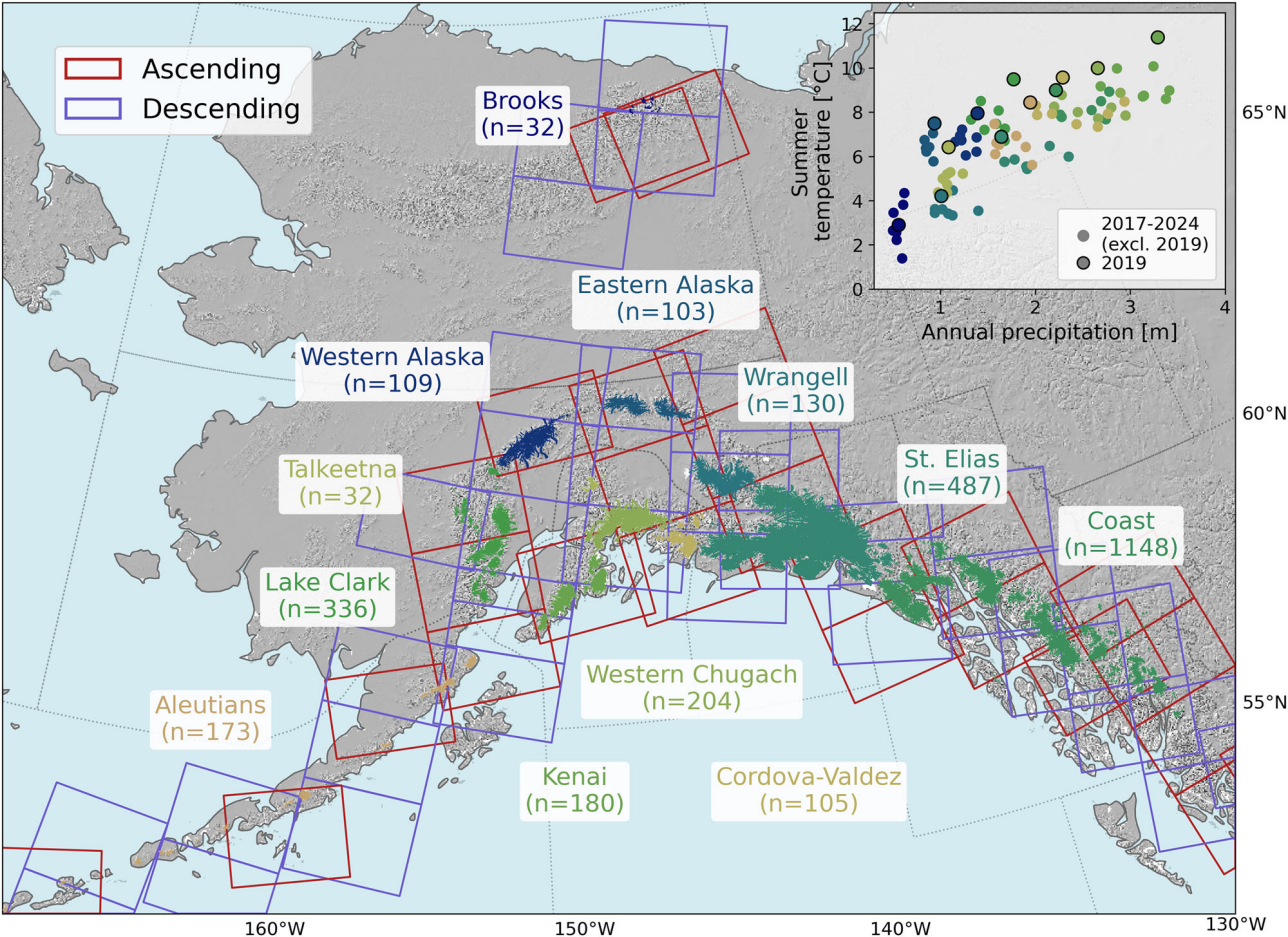
Sentinel-1 coverage of glaciated subregions in Alaska for ascending and descending satellite passes. The number of glaciers greater than 2 km^2^ with Sentinel-1 data are denoted for the twelve subregions of Alaska. Inset shows mean annual precipitation and summer temperature at the terminus of all studied glaciers for each subregion from 2017 to 2024, and highlights those from 2019 associated with a heat wave. The colors on the map and in the inset correspond to each of the subregions.

We estimate melt extents and snowlines from mid-2016 through 2024 for 3023 glaciers in Alaska that are greater than 2 km^2^ using Radiometrically Terrain Corrected Sentinel-1 C-band SAR backscatter data (“Methods”). Sentinel-1 has a 12-day repeat and two orbiting satellites (one of these satellites failed in December 2021), yielding a dense time series of ascending and descending satellite passes over glaciers in Alaska (Fig. 1). Melt extents and snowlines were generated from spatially-distributed backscatter data (Supplementary Fig. 1) and include uncertainty for each observation. To account for SAR incorrectly classifying melt in late summer due to the delayed refreezing process of water in the firn despite sub-freezing climate conditions, we apply an end-of-summer cutoff using bias-corrected temperature reanalysis data^52^. To enable comparison across glaciers of various sizes and elevations, we estimate glacier melt days for each glacier, which represent the cumulative fraction of the glacier area that has melted over time (e.g., one melt day represents the full glacier melting for a day or two days with half the glacier melting) (“Methods”).

**Fig. 2:**
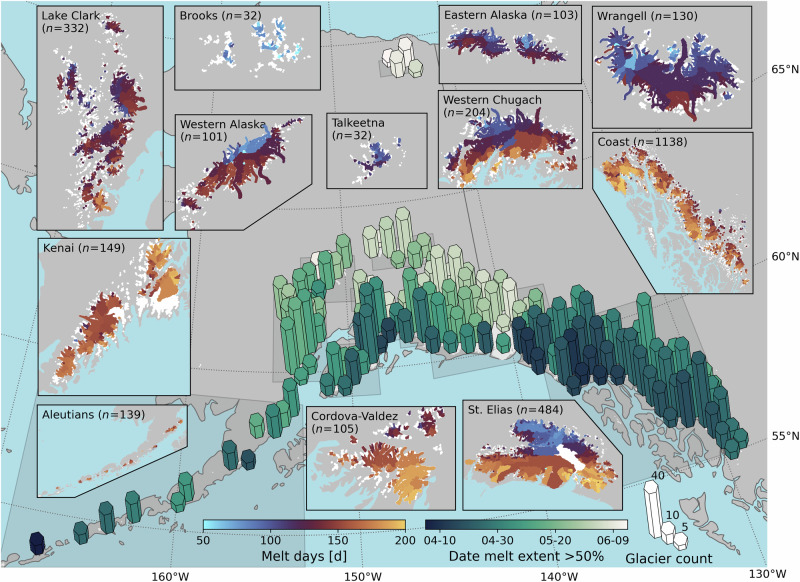
Mean glacier melt days and date when glacier melt extent exceeds the median glacier elevation across Alaska. Hex bar heights indicate the number of glaciers in the area and are colored by the date when the melt extent exceeds 50% of the glacier area. Inset panels show the mean glacier melt days from 2017 to 2024 for each subregion. Data are shown for descending scenes to represent melt that does not refreeze overnight. Data for ascending scenes are shown in the supplementary materials (Supplementary Fig. 3).

Glacier melt varies considerably across Alaska, with more melt along coastal ranges than the interior (Fig. 2, Supplementary Figs. 2 and 3). The ablation season, defined here as the date at which the melt extent exceeds the glacier’s median elevation, begins in early April in the Coast Mountains and Aleutians, which experience a relatively warm and wet maritime climate. In the colder, drier interior, such as the Alaska Ranges or the Brooks Range, melt begins in late May or June (Supplementary Fig. 4). Generally, melt onset coincides with air temperature exceeding 0 °C (Supplementary Fig. 5), the timing of which varies by two months across Alaska. Naturally, the spatial variability of melt (glacier melt days) across Alaska mimics the timing of the melt onset. Across southern Alaska—the Coast Mountains, Cordova-Valdez, Kenai, and Aleutian ranges—glaciers experience up to ~200 glacier melt days each year. Conversely, glaciers in continental climates, including the Brooks, Eastern Alaska, and Wrangell ranges, experience an average of ~50 to 120 glacier melt days each year.

Within subregions, strong patterns of melt exist on the coastal and continental sides of mountain ranges. This is particularly evident in the Eastern Alaska, Wrangell, St. Elias, and Western Chugach ranges, where glaciers on the coastal side experience ~20 more melt days and a melt season up to three weeks longer than their continental counterparts (Supplementary Tables 1 and 2). This is likely due to a combination of climate (the continental side of these ranges in Alaska are generally colder and drier; Supplementary Table 1) and solar aspect (the continental glaciers are generally north-facing and thus receive less direct shortwave radiation).

The timing of satellite passes can provide insight into melt-refreeze cycles^53,54^ as ascending satellite passes are in the evening and descending satellite passes are in the morning for Alaska. The differences in detected melt extents from ascending and descending SAR highlight important physical processes occurring on glaciers by quantifying the timing and length of the shoulder seasons when the climate transitions between the accumulation and ablation seasons. Most subregions demonstrate diurnal melt-refreeze cycles that result in the melt season for descending (morning) passes appearing to be delayed by up to three weeks and having 7–21 fewer glacier melt days (Supplementary Tables 2 and 3). This discrepancy is greatest in the Lake Clark, Eastern Alaska, and Wrangell ranges due to the slow onset of the ablation season, characterized by modest daily melt occurring at the beginning of the ablation season^55,56^, which is refrozen in the descending (morning) backscatter signals. Overall, melt extents derived from ascending and descending SAR thus provide valuable insight into the spatial and temporal variations in diurnal melt-refreeze cycles revealing where the total melt is less than the refreeze capacity of the seasonal snowpack.

### Regional transient snowlines

**Fig. 3:**
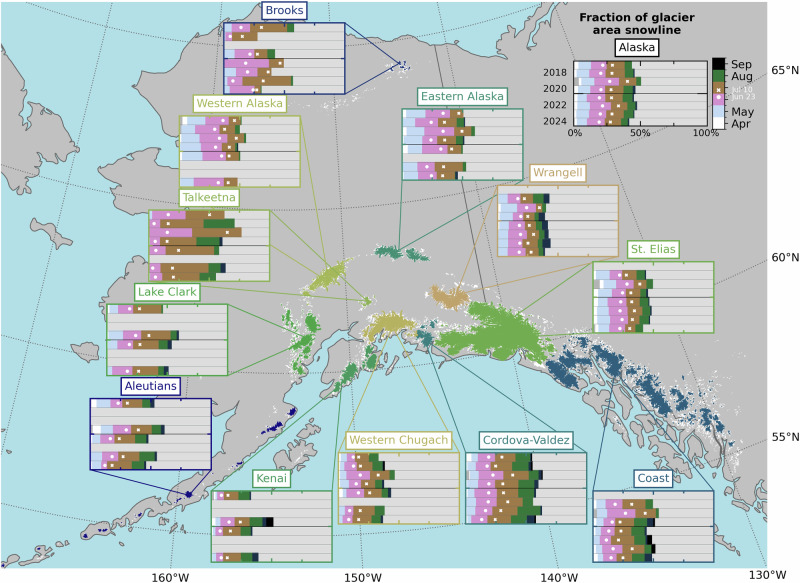
Transient snowline evolution as a fraction of glacier area from 2017 to 2024 for twelve subregions across Alaska. The symbols indicate approximate dates of the start (June 23) and end (July 10) of the 2019 heat wave in Alaska, and the colors correspond to the maximum snowline during each month throughout the summer. Years without snowlines denote data gaps due to insufficient coverage in a given year.

To facilitate regional assessments of transient snowlines and comparisons between glaciers, snowlines are represented as the fraction of total glacier area (Fig. 3). Note that some data gaps exist in the regional analysis—primarily due to missing SAR observations during summer months—as only glaciers with sufficient observations in a given year are considered (“Methods”). In general, the maximum snowline hovers around 50% of total glacier area for all subregions, and there is no clear trend in the maximum annual snowline from 2017 to 2024. While all subregions demonstrate some degree of interannual variability in the maximum snowline, distinct patterns in the timing of snowline retreat are prevalent across all subregions, which is especially evident after extreme events.

The increased snowline retreat in June and July of 2019 is particularly striking. The timing of this retreat coincides with an extreme heat wave from June 23 to July 10, 2019^57^ that extended throughout all of the glaciated subregions of Alaska except the Brooks Range, which is located much further north. In these subregions, the heat wave caused up to a 28% increase in snow-free glacier area in 2019 compared to other years (2017–2024) (Supplementary Table 4, Supplementary Figs. 6 and 7). In a typical year, the snowline would not retreat this high until up to two months later (Supplementary Fig. 8). This change in the timing of snowline retreat and lengthened exposure of bare ice and firn reduces the glacier albedo, affecting the glacier energy balance and increasing mass loss^58^.

Unlike the melt extents, glacier snowlines are consistent on the coastal and continental sides of mountain ranges (Supplementary Figs. 9 and 10). This indicates a major difference in accumulation patterns across these mountain ranges, as differences in accumulation are the only way snowlines could remain consistent across coastal and continental glaciers, despite the differences in observed melt days. This implied complexity in subregional melt and accumulation highlights the importance of accurate high spatial-resolution climate data and observational constraints for modeling remote glaciers.

### Climate impacts on melt extents and snowlines

**Fig. 4:**
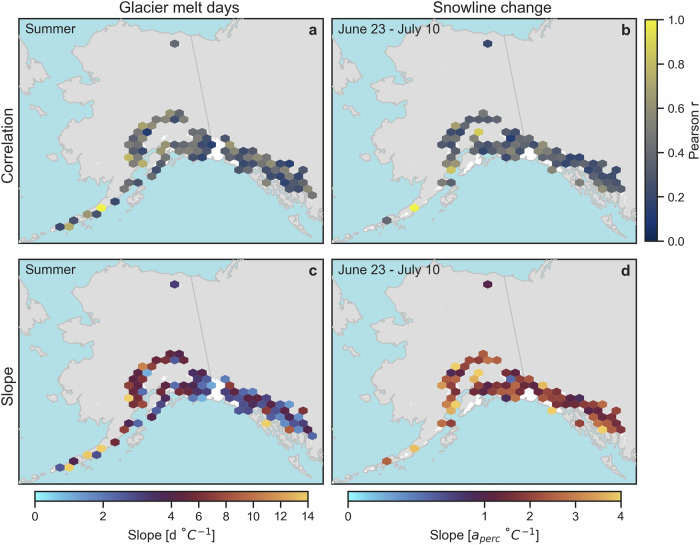
Relationship between glacier melt days and summer temperatures as well as snowline changes and mid-summer temperatures. Correlation and slope between glacier melt days and summer (May through September) temperatures (**a**, **c**) are based on each glacier and each year. Relationships for snowline change up to July 10 and temperatures from June 23 to July 10, coincide with the dates of the 2019 heat wave (**b**, **d**). Data represent all observations within a given area. Only pixels with a significant correlation (*p* < 0.05) are shown.

We assess the impact of climatic forcing by evaluating relationships between melt extents and transient snowlines with temperature and precipitation data. Specifically, we evaluate the correlation between derived glacier metrics (melt days, maximum snowline, and snowline changes) and climatic variables (temperature and precipitation) over various monthly and seasonal periods. While both temperature and precipitation were evaluated, only temperature is strongly correlated with any of the glacier metrics. Significant (*p* < 0.05) correlations exist between glacier melt days and summer temperatures (May through September) across glaciers in all subregions of Alaska (Fig. 4; Supplementary Table 5; Supplementary Fig. 11). In particular, glaciers across various parts of the Aleutians and Lake Clark subregions experience up to an additional 2 weeks of glacier melt days per 1 °C summer warming. This likely reflects the combination of long transition seasons (Supplementary Table 3), which increase the sensitivity of glacier melt days to small temperature changes, and large interannual variability in summer temperatures (Fig. 1) in these subregions. Weaker correlations between melt days and summer temperatures occur in the Kenai and Coast Mountains, likely due to the early onset of melt, which begins in April, such that additional summer warming does not substantially contribute to additional melt extent, since the glaciers are already experiencing widespread melt by May (Fig. 2). Correlations are slightly less dramatic for other subregions, although they generally show 2–7 additional glacier melt days per 1 °C of summer warming. For reference, 2019 summer temperatures on glaciers in these subregions were 0.8 to 2.0 °C warmer than the 2017–2024 (excluding 2019) mean summer temperature (Supplementary Table 6). This suggests that every fraction of a degree of summer warming has a significant effect on the glaciers causing up to a month of additional glacier melt days.

While no significant relationships were found with the maximum snow-free area, high snowlines after the 2019 heat wave (which occurred from June 23 to July 10) were significantly correlated with temperature. Specifically, each additional 1°C warming corresponds to a 1–4% increase in exposed ice area fraction (Fig. 4; Supplementary Table 5; Supplementary Fig. 12). Given the 2019 heat wave temperatures were 2.1 to 6.8°C warmer than the typical year (Supplementary Table 6), this relationship highlights the ability of SAR to capture the impact of the heat wave on the glaciers and, more importantly, quantifies the sensitivity of glaciers to heat waves. While subregional snowlines increased by up to 105 m during the heat wave, exposing up to 28% more ice during the 2019 heat wave compared to the same time period in other years (Supplementary Table 4), snowlines on individual glaciers increased by more than 240 m, exposing up to an additional 33% of glacier area (Supplementary Fig. 13).

## Discussion

**Fig. 5:**
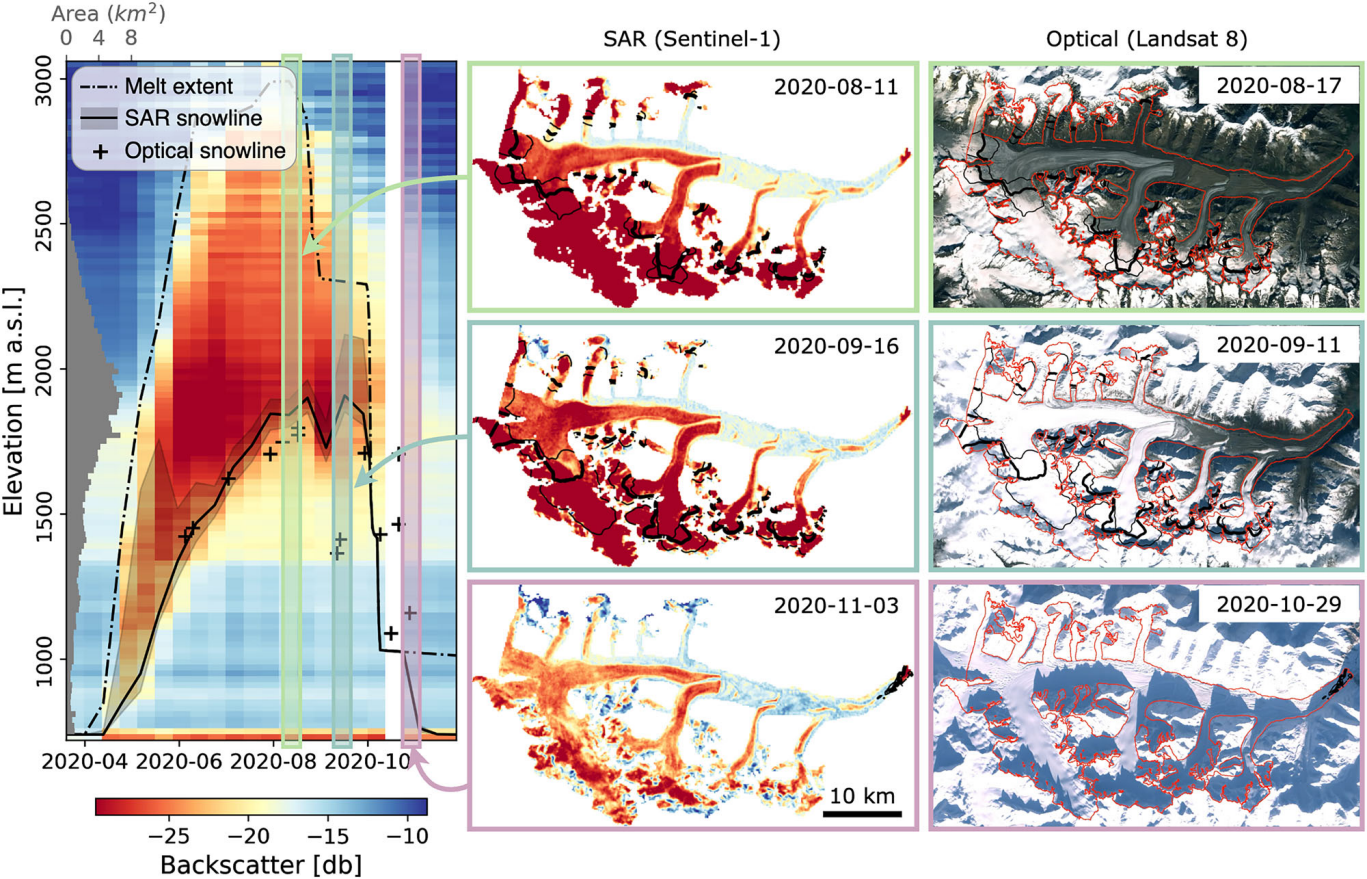
Example of snowlines and melt extents derived from SAR compared to optical snowlines (ref. ^22^) on Black Rapids Glacier in 2020. Glacier hypsometry is shown on top of the heat map. SAR acquisitions and corresponding snowlines are shown in the middle panels (generally appearing just below the dark red areas, where thinner lines represent snowline uncertainty). The nearest optical imagery with limited cloud coverage are shown on the right, with the SAR-derived snowline from the middle panel overlaying the images. Landsat-8 imagery (right column) courtesy of the U.S. Geological Survey.

In this paper, we leverage SAR to present a new dataset of transient glacier snowlines and melt extents across Alaska, and assess glacier changes relative to climatic factors. To our knowledge, we provide the first large-scale, systematic application of SAR to derive transient glacier snowlines. Glacier transient snowline altitude time series show strong agreement with existing datasets derived from optical sources (r^2^ up to 0.94), indicating the suitability of Sentinel-1 SAR to obtain accurate snowlines (Supplementary Text S1, Supplementary Table 7, Supplementary Figs. 14–25). Some discrepancy between snowlines detected from SAR and optical sources is expected, particularly after snowfall events, since SAR penetrates dry snow and observes the underlying surface. Consequently, SAR-derived snowlines may lag in detecting descending snowlines or fail to capture minor snowfall events (Fig. 5). However, this provides SAR with the unique and powerful ability to still observe the maximum snowline altitude (i.e., the equilibrium-line altitude) even when satellite acquisitions occur after new snowfall that has not yet melted.

SAR also offers powerful advantages to snowlines derived from optical sources^21–24,32^ as the methods require no training or calibration, are robust to climate and daylight (including cloud cover, shadows, shading, and general changes in lighting conditions), and obtain measurements on a 6 to 12-day basis. As such, snowlines from SAR produce regular snowline observations throughout the entire ablation season, including in shoulder seasons where snowlines from optical sources are the least reliable. This consistency is particularly valuable towards the end of the summer, as snowlines are often used to estimate the equilibrium-line altitude. Like optical data, distinguishing firn from snow is more challenging than snow from ice. However, our results show that the change in backscatter signal from snow to firn is substantial enough to estimate the snowline above the equilibrium-line altitude as accurately as optical data (Supplementary Text S1). Furthermore, unlike many existing snowline products, our workflow quantifies uncertainty in transient snowline observations, which is critical for utilizing snowlines as calibration for models at subseasonal resolution^26^. Ultimately, these data present unique opportunities for near real-time monitoring of glaciers and serve as a foundation to augment existing regional and global datasets by providing subseasonal data at the glacier scale^4,59,60^.

Our new datasets estimate transient melt extents and snowlines with unprecedented reliability. These findings provide insights into the ways in which Alaskan glaciers are responding to climate change, and have implications at local, regional, and global scales. The present-day sensitivity to warming indicates that glaciers across Alaska will experience up to 1 to 4 weeks of additional glacier melt days by the end of the 21st century based on present-day glacier areas, depending on the emissions scenario (Supplementary Table 5, Supplementary Fig. 26). Similarly, mid-summer snowlines will continue to be susceptible to heat waves, however, the climate even in typical years will cause snowlines to retreat earlier and expose ~5% more ice regionally through mid-summer by 2100, depending on the future emissions scenario (Supplementary Table 5, Supplementary Fig. 27). The open-source methods and accompanying analyses are well suited to scale to other regions and/or globally, which would open unique opportunities to leverage the data as calibration and validation for temperature reanalyses and global glacier models. Such assessments will constrain models at subseasonal scales, thereby improving estimates of future glacier changes and contributions to sea-level rise.

## Methods

### Data overview and preparation

SAR data was obtained via the Alaska Satellite Facility (ASF) Vertex search interface (https://search.asf.alaska.edu/) for all satellite path-frame combinations covering glaciated terrain over Alaska and that had at least 100 scenes between January 2016 and December 2024. Radiometrically Terrain Corrected (RTC)^61^ SAR ground range detected high-resolution data (GRD-HD) integrated waveform (IW) products were downloaded at 30 m pixel spacing using γ_0_ backscatter coefficient normalization and a decibel output scale. DEM matching was applied to effectively co-register SAR images to the Copernicus Global 30 m DEM^62^. Both Sentinel-1A and Sentinel-1B data were utilized. In total, we considered 50 path-frame combinations: 18 from ascending satellite passes and 32 from descending satellite passes, each with between 102 and 248 scenes.

Cross-polarized (VH) backscatter products were extracted to improve sensitivity to volume scattering of the radar signal in firn and snow and more effectively detect changes in glacier facies compared to co-polarized data^63^ (Supplementary Fig. 25). All data from each path-frame were aggregated into a single time series datacube and downscaled from 30 m to 100 m resolution (Supplementary Fig. 24), with observations every ~12 days since mid-2016. We extracted data for each glacier in a scene with an area of at least 2 km^2^, representing 99% of glaciers in Alaska that have an area of at least 2 km^2^, and 85% of the total glaciated area in Alaska.

### Melt extent altitude

Glacier melt extents reveal where the glacier is melting for each scene. The melt extent is characterized by a sharp drop in backscatter due to the presence of water in the snowpack that absorbs and attenuates the radar signal^36^. The initial classification of melting is based on a time series analysis of backscatter relative to the winter mean for each pixel (Supplementary Fig. 28). Specifically, pixels are classified as melting when the backscatter decreases by at least 3 dB (a ~50% decrease in power) compared to the mean winter backscatter^37^ and the change in backscatter is greater than twice the winter standard deviation^40^. The winter mean is based on scenes from January and February. Pixels with a standard deviation from winter scenes that exceeds 3 dB are excluded as the backscatter should be relatively stable during this time. Since pixels are often misclassified as not melting when the surface becomes snow-free and the backscatter no longer has the marked drop (see snowlines below), a second step classifies these snow-free pixels as melting when the higher, snow-covered pixels are still melting. Specifically, we aggregate the melt pixels to 20 m elevation bins, identify the minimum elevation bin with at least 10 pixels of which 90% are melting, and classify all elevations below this bin as melting.

The melt extent altitude is derived by estimating the percent of a glacier’s pixels that are classified as melting and determining the corresponding elevation from the cumulative area altitude distribution^24^. For example, if 50% of the glacier is melting, the melt extent altitude is the glacier’s median elevation. This percentage-based method is robust to complex glacier geometries with multiple tributaries and melt extents that vary considerably spatially. Uncertainty is calculated from the corresponding elevations associated with the percentage of pixels that are classified as melting but above the melt extent and, vice versa, that are classified as not melting but are below the melt extent.

### Transient snowline altitude

Transient snowline altitudes are estimated using a similar percentage-based approach as melt extent altitudes. Snow-free glacier pixels are determined as those that are below the melt extent, have a 4 dB increase (a ~2.5x increase in power) compared to the 5th percentile of summer (April, May, June, and July) backscatter, and have a change in backscatter exceeding at least twice the winter pixel standard deviation (Supplementary Fig. 28). The 5th percentile of minimum summer backscatter effectively captures a point in the time series that has the characteristic drop in backscatter associated with snow melting on the surface. The percentage of snow-free pixels on the glacier is then used to estimate the transient snowline altitude based on the corresponding elevation from the cumulative area altitude distribution. Uncertainty is estimated as the elevation associated with anomalous snow-covered and snow-free pixels below and above the transient snowline altitude, respectively.

### End-of-summer cutoff

Melt extent altitudes are unreliable in the late summer due to a lag between actual surface conditions (i.e., no melt) and the delayed refreezing within the snow/firn that dictates SAR backscatter signals. As such, a drop in SAR backscatter can remain in accumulation areas, specifically the wet percolation zone, even after the ablation season has ended and the glacier is no longer melting. We use daily air temperature data from the European Centre for Medium-Range Weather Forecasts (ECMWF) Reanalysis v5 (ERA5)^52^ to apply an end-of-summer cutoff for the melt extent altitudes. We bias-correct the temperature data using an additive temperature factor that minimizes the misfit between SAR-derived melt onset and the initial date when temperature exceeds 0°C for each elevation bin. Using a lapse rate of 6.5 °C km^−1^, we adjust the bias-corrected ERA5 temperature time series to each glacier elevation bin, and apply an end-of-summer cutoff to the melt extent altitudes for each elevation bin based on the final date with above-freezing temperatures.

Transient snowline altitudes at the end of summer are also adjusted to ensure that the transient snowline altitude does not exceed the melt extent altitude. We note that this may incorrectly lower snowlines after the ablation season (e.g., October and November) once melting has stopped but if snow has not yet fallen; however, this has a negligible impact on our results as the focus of our study is on the rising or maximum snowline over the ablation season.

### Glacier change metrics

We use “glacier melt days” as a normalized metric representing the duration and spatial extent of glacier melt each year. Specifically, glacier melt days are calculated annually from the melt extent altitude time series, representing the number of days during which the equivalent of the glacier’s total area is melting (e.g., one melt day represents the full glacier melting for a day or two days with half the glacier melting). The date of melt onset is calculated as the date in which the melt extent exceeds 50% of the glacier area. This threshold is chosen as it is robust to noise and approximately coincides with the glacier accumulation area experiencing melt.

### Snowline validation

The transient snowline time series derived from this study are compared to existing studies that report snowlines or ELA for glaciers in Alaska^21–24,32^. We report metrics including the correlation coefficient, mean error (bias), mean absolute error, and root mean square error. For datasets that report only an annual ELA (i.e., ref. ^23^), the maximum annual snowline altitude is taken from the SAR-derived snowline as the ELA. For datasets that report snowline altitudes for specific dates, the SAR-derived transient snowline time series are linearly interpolated to obtain a snowline altitude estimate for the corresponding date. SAR-derived snowline data are only compared if there are at least 20 SAR observations for a given year, 10 of which occur from May through September.

### Climate analyses

To assess the impact of climate on glaciers, temperature and precipitation data were correlated with melt extent altitudes, transient snowlines, and glacier change metrics. Hourly ERA5 temperature and precipitation data were aggregated to daily values (mean and cumulative, respectively). The Pearson linear correlation coefficient and slope of correlations were calculated for various time spans throughout the summer. We report ‘significant’ correlations using a Wald test (*p* < 0.05). Correlations were extracted between glacier metrics (i.e., glacier melt days and snowline changes) and climatic variables (i.e., temperature and precipitation for a given time span) for each SAR path per glacier and year. The reported correlations are the results of glaciers aggregated by subregion (e.g., Supplementary Table 5, Supplementary Figs. 11 and 12) or locally into hex bins with neighboring glaciers (e.g., Fig. 4). Projections of temperatures through 2100 are from an ensemble of 12 general circulation models (GCMs) from the Coupled Model Intercomparison Project Phase 6 (CMIP6) and four shared socioeconomic pathways (SSPs), using an 11-year moving average to filter through interannual variability.

## Supplementary information

Supplementary Information

## Data Availability

All data produced in this work are available with open access, and the source data are publicly accessible. Sentinel-1 SAR data can be downloaded through the Alaska Satellite Facility Vertex tool (https://search.asf.alaska.edu/#/). Glacier outlines are available online through the Global Land Ice Measurements from Space (GLIMS) initiative (https://www.glims.org/RGI/). Climate data are available online at the Copernicus Climate Change Service (C3S) Climate Data Store (cds.climate.copernicus.eu/). All glacier transient snowlines, melt extents, and binned backscatter products are available from Zenodo (https://zenodo.org/records/17108203) and can be easily accessed, visualized, and downloaded for any glacier through an online tool (https://alaskasnowlines.streamlit.app/).

## Code availability

Python was used to generate all results and analysis in this study. Information regarding Python installation and system requirements is available online (https://www.python.org/). Code used to produce these results and generate figures is available on GitHub (https://github.com/albinwwells/SAR-Alaska-Processing) with a sample dataset and accompanying tutorial available on Zenodo (https://zenodo.org/records/17108203).
