# Supplementary material for: Seasonal progression of melt and snowlines in Alaska from SAR reveals impacts of warming
Source: NPJ Clim Atmos Sci. 2026 Feb 4;9(1):95. doi: 10.1038/s41612-026-01321-y (PMC13065479; doi:10.1038/s41612-026-01321-y)
Supplement: Supplementary file 1 — Supplementary Information [file 41612_2026_1321_MOESM1_ESM.pdf]

Supplementary Material

Seasonal progression of melt and snowlines in Alaska from SAR reveals impacts of warming

Albin Wells*, David R. Rounce, and Mark Fahnestock

*** Correspondence:** Albin Wells: awwells@cmu.edu

TABLE OF CONTENTS

1. Supplementary Text
- **Supplemental text S1:** SAR-derived snowlines comparison to existing datasets *(Page 3)*

2. Supplementary Tables
- **Table S1:** Melt days for coastal and continental glaciers *(Page 4)*
- **Table S2:** Melt season start, end, and length for coastal and continental glaciers *(Page 5)*
- **Table S3:** Glacier melt days for ascending and descending satellite passes *(Page 6)*
- **Table S4:** Subregional snowline changes during heat wave period *(Page 7)*
- **Table S5:** Subregional correlation between glacier change and climate *(Page 8)*
- **Table S6:** Temperature anomaly over summer 2019 *(Page 9)*
- **Table S7:** Validation of SAR-derived transient snowlines *(Page 10)*

3. Supplementary Figures
- **Figure S1:** Example of spatially-distributed and binned SAR backscatter *(Page 11)*
- **Figure S2:** Annual glacier melt days and melt season start across Alaska *(Page 12)*
- **Figure S3:** Glacier melt days and date of melt onset across Alaska for ascending scenes *(Page 13)*
- **Figure S4:** Map of melt season start and end *(Page 14)*
- **Figure S5:** Melt extent and temperature for Alaska benchmark glaciers *(Page 15)*
- **Figure S6:** Transient snowline from 2017-2024 for select Alaska glaciers *(Page 16)*
- **Figure S7:** Snowline changes resulting from the 2019 heat wave *(Page 17)*
- **Figure S8:** Time to reach 2019 end-of-heat wave snowline for 2017-2024 *(Page 18)*
- **Figure S9:** Maximum snowline date and area fraction across Alaska for descending scenes *(Page 19)*
- **Figure S10:** Maximum snowline date and area fraction across Alaska for ascending scenes *(Page 20)*
- **Figure S11:** Correlation between glacier melt days and summer temperature *(Page 21)*
- **Figure S12:** Correlation between snowline change and temperature during 2019 heat wave time period *(Page 22)*
- **Figure S13:** Histogram of heat wave impact on glacier snowline change *(Page 23)*
- **Figure S14:** Snowline comparison to Larocca et al., 2024 *(Page 23)*

- **Figure S15:** Snowline comparison to Aberle et al., 2025a *(Page 24)*
- **Figure S16:** Transient snowlines from SAR and Aberle et al., 2025a *(Page 25)*
- **Figure S17:** Snowline comparison to Aberle et al., 2025b *(Page 26)*
- **Figure S18:** Snowline comparison to Aberle et al., 2025b prior to September *(Page 27)*
- **Figure S19:** Snowline comparison to Bevington & Menounos, 2025 *(Page 28)*
- **Figure S20:** Snowline comparison to Bevington & Menounos (2025) for June, July, and August *(Page 29)*
- **Figure S21:** Example snowline comparison to Bevington & Menounos, 2025 *(Page 30)*
- **Figure S22:** Maximum annual snowline comparison to Zeller et al., 2025 *(Page 31)*
- **Figure S23:** Maximum annual snowline from Bevington and Menounos (2025) comparison to Zeller et al., 2025 *(Page 32)*
- **Figure S24:** Example of SAR backscatter at 30 m and 100 m resolution *(Page 33)*
- **Figure S25:** Example backscatter from cross-polarized and co-polarized SAR *(Page 34)*
- **Figure S26:** Subregional warming for summer months from 2000-2100 *(Page 35)*
- **Figure S27:** Subregional warming for June and July from 2000-2100 *(Page 36)*
- **Figure S28:** Sample pixel backscatter and delineated melt and snow areas *(Page 37)*

4. Supplementary Material References

1 SUPPLEMENTARY TEXT

Supplemental text S1: SAR-derived snowlines comparison to existing datasets

We compare the transient snowlines derived from this study with numerous existing datasets that systematically calculate snowlines in Alaska[1-5]. Standard error metrics (mean error, mean absolute error, and correlation) are used to quantify agreement, although we importantly note that the optical datasets are susceptible to considerable errors and thus cannot be considered ground truth. The analyses thus focus on highlighting the suitability of SAR to estimate snowlines compared to optical datasets as ground truth observations are not available.

Comparisons with late-summer snowlines produced for 11 glaciers greater than 2 km^2 in Alaska from Larocca et al. (2024)[1] show a mean error of -45 m and mean absolute error of 86 m (r^2=0.94) for all observations from 2017-2022 (Supplementary Fig. 11). Validation against transient snowlines for three benchmark glaciers in Alaska from automated methods (ref. 3) show substantial noise in the optical dataset–which has frequent and clearly erroneous snowline spikes and dips–that encumber comparisons of transient snowline time series, although SAR-derived snowlines generally appear within the noise of the optical dataset (Supplementary Figs. 12 and 13). We further compare results to Aberle et al. (2025b)[4] for an expanded set of 67 glaciers in Alaska from 2017-2023, which shows some bias (148 m) but general agreement (r^2=0.76) between SAR-derived snowlines and reported monthly snowline observations (Supplementary Fig. 14). We note that agreement is very high when comparing only observations prior to September (mean error of 91 m, r^2=0.87) (Supplementary Fig. 15).

On a regional level, our transient snowlines agree well with data produced from optical sources (ref. 5), especially prior to September (Supplementary Figs. 16 and 17). SAR-derived snowlines show minimal bias in June, July, and August (mean error of 6 m, r^2=0.64 for 216,975 observations) and throughout the entire summer (mean error of 59 m, r^2=0.79 for 337,897 observations) compared to 2017-2024 snowlines from Bevington and Menounos (2025)[5]. The largest discrepancies between the datasets arise from clear outliers in optical data (Supplementary Fig. 18). We note that late summer snowlines above the equilibrium-line altitude are challenging to delineate in optical datasets[6], which is similarly the case for SAR data. However, our results indicate that despite the change in SAR backscatter from snow to firn being less clear than the change from snow to ice, the change in backscatter signal from snow to firn is substantially different enough to estimate the snowline above the equilibrium-line altitude as accurately as optical data. Similar comparisons with equilibrium-line altitudes for 11,099 observations from 2018-2022 show a mean error of -88 m (r^2=0.67) (ref. 2), which is comparable to the agreement between 6,872 observations in Bevington and Menounous (2025)[5] and Zeller et al. (2025)[2] (mean error of -57 m, r^2=0.68) (Supplementary Figs. 19 and 20).

Overall, we find that SAR is suitable for deriving transient snowlines throughout the entire ablation season, and has the unique ability to detect the end-of-summer snowline even if the satellite acquisition occurs after the end of the ablation season as SAR penetrates dry snow (Fig. 5). For automated detection, snowlines generated from SAR contain less noise than optical

datasets as SAR is robust to climate and lighting conditions. Finally, the transient snowlines produced in this study have quantified uncertainty bounds for each observation (Methods), which is another benefit over many existing datasets.

2 SUPPLEMENTARY TABLES

Table S1: Mean 2017-2024 glacier melt days and key climate variables on the coastal and continental sides of each mountain range. Glaciers are categorized as being on the coastal or continental side of a mountain range using an aspect cutoff for each subregion. Melt days are the mean values from ascending and descending scenes. Count refers to the number of glaciers with SAR data on each side of the respective mountain range. Temperature and precipitation are quantified from the nearest ERA5 grid cell for each glacier with SAR data, and averaged for these glaciers over the respective time period. We apply a lapse rate of -6.5 ˚C/km to adjust temperature data to the glacier terminus.

Subregion	Coastal aspect range [deg]	Coastal				Continental				Melt days coastal minus continental [d]
		Melt days [d]	Count	Summer temp. [˚C]	Annual precip. [m]	Melt days [d]	Count	Summer temp. [˚C]	Annual precip. [m]	
Aleutians	160 - 340	163.8	79	6.72	1.78	163.8	73	6.81	1.70	0.0
Brooks	90 - 270	84.4	1	1.97	0.57	77.5	31	2.95	0.56	6.8
Coast	130 - 310	167.6	299	7.84	2.69	157.1	844	8.43	2.35	10.6
Cordova-Valdez	130 - 310	169.0	31	8.07	2.81	154.2	74	8.12	2.24	14.8
Eastern Alaska	100 - 280	139.3	43	6.21	0.94	120.5	60	6.81	0.90	18.8
Kenai	30 - 210	171.1	56	10.02	3.34	169.2	123	9.20	3.05	1.9
Lake Clark	10 - 190	141.6	122	8.18	1.59	135.0	214	7.57	1.56	6.6
St. Elias	90 - 270	154.1	107	6.03	2.38	133.9	374	5.94	1.74	20.2
Talkeetna	90 - 270	123.8	2	4.58	1.21	109.1	30	5.10	1.07	14.7
Western Alaska	70 - 250	138.2	21	6.79	1.30	128.0	66	6.84	1.27	10.2
Western Chugach	90 - 270	167.5	45	9.69	3.08	141.8	133	8.09	2.47	25.7
Wrangell	90 - 270	124.3	41	4.28	1.18	106.3	89	3.43	1.04	18.0

Table S2: Melt season start, end, and length for coastal and continental sides of each Alaska subregion from 2017-2024. Melt season start and end is estimated as the first and last date where melt extent exceeds 50% of the glacier elevation range for ascending and descending scenes.

Subregion	Melt season start [DOY]				Melt season end [DOY]				Melt season length [d]			
	Ascending		Descending		Ascending		Descending		Ascending		Descending	
	Coast	Cont	Coast	Cont	Coast	Cont	Coast	Cont	Coast	Cont	Coast	Cont
Aleutians	115.0	114.3	118.9	118.7	297.4	298.2	292.3	290.9	182.4	183.8	173.4	172.2
Brooks	145.3	153.9	158.8	158.6	243.0	236.4	235.4	236.8	97.7	82.5	76.5	78.2
Coast	107.4	114.8	112.8	119.1	290.4	284.9	291.3	285.1	183.0	170.1	178.5	166.0
Cordova-Valdez	108.2	115.6	116.8	126.7	296.2	284.5	293.1	282.1	188.0	168.9	176.3	155.4
Eastern Alaska	123.4	137.5	139.6	147.8	274.8	263.1	265.5	254.5	151.4	125.6	125.9	106.6
Kenai	104.9	107.1	117.4	121.0	294.7	293.5	300.9	294.9	189.8	186.3	183.5	173.9
Lake Clark	112.3	113.9	133.0	135.2	274.2	274.0	274.2	272.9	161.9	160.0	141.2	137.7
St. Elias	113.6	128.0	125.7	137.3	277.5	274.1	281.2	270.8	163.9	146.1	155.5	133.4
Talkeetna	–	137.4	140.2	150.2	–	254.4	265.8	259.7	–	117.0	125.6	109.4
Western Alaska	121.8	134.4	136.7	145.2	270.2	264.7	268.1	261.4	148.4	130.3	131.4	116.3
Western Chugach	110.9	122.0	116.9	133.2	287.6	280.6	289.2	274.6	176.6	158.7	172.4	141.4
Wrangell	134.4	143.8	146.2	154.4	271.4	262.6	260.9	255.7	137.0	119.0	114.6	101.3

Table S3: Mean 2017-2024 glacier melt days for ascending and descending satellite passes. Count refers to the number of glaciers with SAR data from ascending and descending satellite passes for each subregion.

Subregion	Ascending		Descending		Ascending minus descending [d]
	Melt days [d]	Count	Melt days [d]	Count	
Aleutians	175.2	85	161.4	145	13.8
Brooks	83.8	26	75.6	32	8.1
Coast	161.5	1132	158.3	1138	3.1
Cordova-Valdez	166.4	105	154.2	105	12.2
Eastern Alaska	138.2	103	119.7	103	18.5
Kenai	175.3	145	163.9	148	11.4
Lake Clark	153.6	335	132.2	332	21.5
St. Elias	146.5	431	135.9	480	10.6
Talkeetna	120.6	2	109.5	32	11.1
Western Alaska	141.5	84	127.1	82	14.4
Western Chugach	158.1	86	143.5	178	14.6
Wrangell	123.1	130	106.6	130	16.5

Table S4: Mean subregional snowline retreat from June 23 to July 10 during the 2019 heat wave and typical years (2017 through 2024). Note, insufficient data exists in 2019 for the Aleutians and Kenai subregions due to mid-summer gaps in Sentinel-1 SAR acquisitions.

Subregion	Snowline area retreat [%]			Snowline elevation retreat [m a.s.l.]		
	2017-2024	2019	Difference	2017-2024	2019	Difference
Aleutians	8.8	–	–	83	–	–
Brooks	15.5	17.9	2.4	119	106	-14
Coast	11.1	18.6	7.5	126	148	22
Cordova-Valdez	10.8	19.6	8.7	112	167	55
Eastern Alaska	13.6	18.6	5.0	147	159	12
Kenai	8.2	–	–	99	–	–
Lake Clark	11.6	27.4	15.8	111	216	105
St. Elias	10.5	19.4	8.9	114	159	45
Talkeetna	12.3	41.0	28.7	120	197	77
Western Alaska	12.7	17.5	4.8	115	158	43
Western Chugach	9.3	18.7	9.3	118	178	60
Wrangell	10.5	15.7	5.2	107	128	21

Table S5: Subregional correlation between melt days or snowline change and temperature. Count refers to the total number of observations (glaciers, years, and SAR paths).

Subregion	Count	Glacier melt days and summer temperature		Snowline area change and heat wave period temperature	
		p-value	Slope [d $^{\circ}C^{-1}$]	p-value	Slope [$a_{percent}$ $^{\circ}C^{-1}$]
Aleutians	509	<0.01	4.2	0.02	0.7
Brooks	172	<0.01	2.7	0.18	0.6
Coast	8604	<0.01	3.2	<0.01	1.2
Cordova-Valdez	946	<0.01	6.6	<0.01	2.0
Eastern Alaska	775	<0.01	5.3	<0.01	1.9
Kenai	509	<0.01	2.6	0.03	0.5
Lake Clark	891	<0.01	5.6	<0.01	1.6
St. Elias	3916	<0.01	6.3	<0.01	1.7
Talkeetna	105	0.09	2.2	<0.01	3.9
Western Alaska	515	<0.01	6.8	<0.01	1.6
Western Chugach	868	<0.01	5.0	<0.01	1.3
Wrangell	1302	<0.01	3.8	<0.01	1.1

Table S6: Temperature difference between 2019 and the mean of all other years (2017-2024) for various time periods for glaciers in each subregion of Alaska.

Subregion	Period						
	May [˚C]	June [˚C]	July [˚C]	August [˚C]	September [˚C]	Summer [˚C]	Heat wave (06/23-07/10) [˚C]
Aleutians	0.96	1.94	2.40	2.90	1.56	1.96	4.47
Brooks	2.37	0.37	1.07	-3.88	1.43	0.26	0.24
Coast	1.85	0.65	1.06	-0.36	0.70	0.78	2.93
Cordova-Valdez	0.51	1.56	2.46	2.10	1.72	1.67	5.49
Eastern Alaska	1.70	1.44	1.50	-0.31	1.86	1.23	3.74
Kenai	0.39	2.25	3.26	3.03	1.04	2.00	6.79
Lake Clark	0.18	2.49	2.91	2.12	1.11	1.76	6.49
St. Elias	1.49	1.23	1.41	0.18	1.54	1.17	3.79
Talkeetna	0.52	1.97	2.48	1.24	1.87	1.61	5.65
Western Alaska	0.48	1.76	1.98	0.67	1.20	1.21	4.57
Western Chugach	0.54	1.88	2.59	1.95	1.56	1.70	5.87
Wrangell	1.88	0.88	0.53	-0.69	1.84	0.80	2.09

Table S7: Validation metrics between SAR-derived transient snowlines and existing datasets. Count refers to the total number of snowline observations used for comparison. Note that the comparison with Bevington and Menounos (2025)[5] is for snowlines in June, July, and August.

Year	Larocca et al. (2024)[1]				Bevington and Menounos (2025)[5]				Zeller et al. (2025)[2]			
	r^2	Bias [m]	RMSE [m]	Count	r^2	Bias [m]	RMSE [m]	Count	r^2	Bias [m]	RMSE [m]	Count
2017	0.96	-65	79	3	0.86	-41	172	4,790	–	–	–	–
2018	0.97	-145	183	4	0.85	-24	183	11,867	0.67	-156	296	2,188
2019	1.00	-32	45	2	0.82	21	197	42,509	0.74	-312	393	2,180
2020	0.84	3	108	7	0.86	14	187	28,162	0.66	-25	258	2,870
2021	0.92	-35	117	9	0.86	6	186	43,804	0.73	40	275	2,771
2022	–	–	–	–	0.87	-8	184	21,670	0.75	1	273	1,090
2023	–	–	–	–	0.86	0	192	33,066	–	–	–	–
2024	–	–	–	–	0.87	11	192	31,107	–	–	–	–
All	0.94	-45	120	25	0.86	6	189	216,975	0.67	-88	302	11,099

3 SUPPLEMENTARY FIGURES

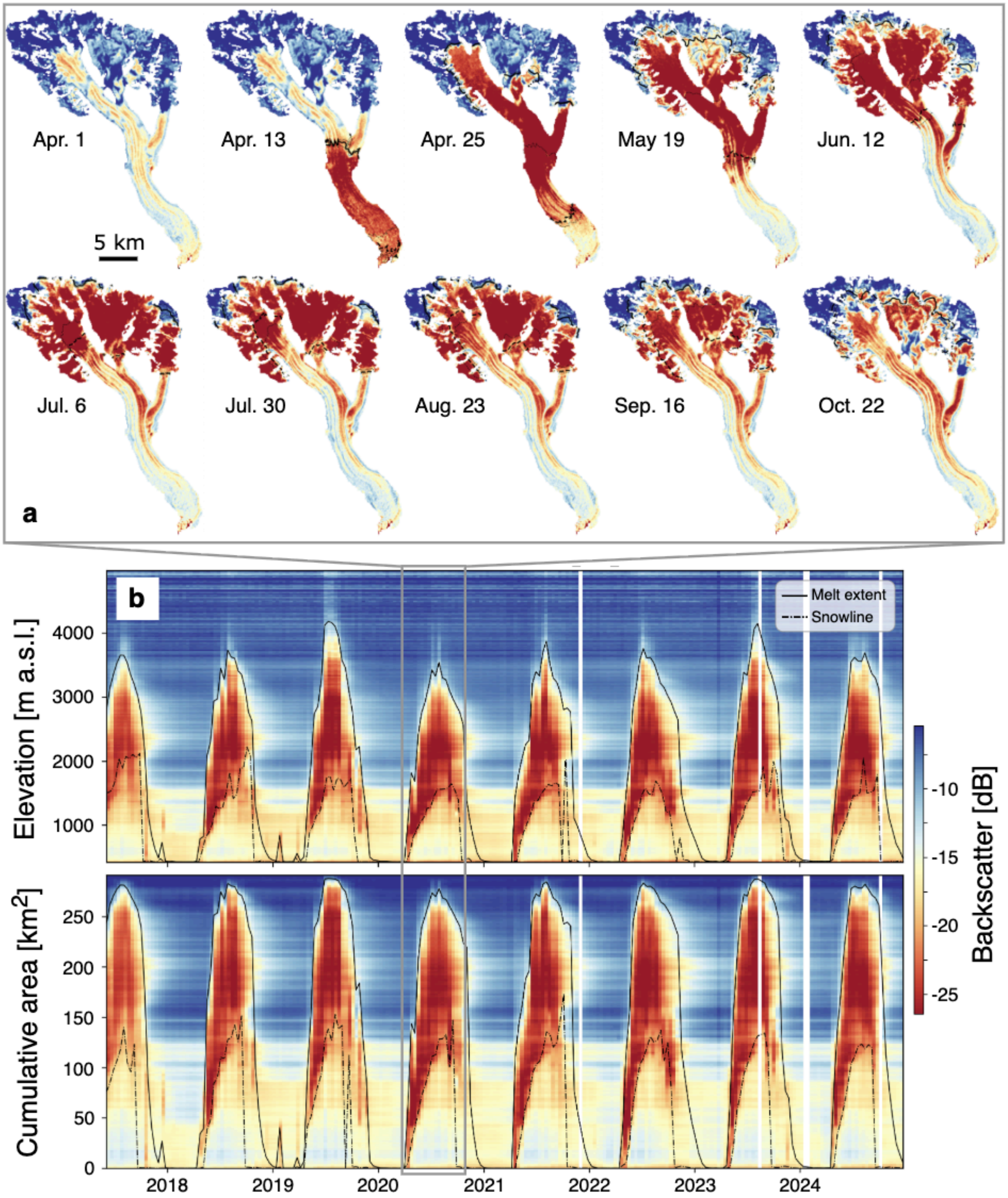

Fig. S1: (a) Spatially-distributed SAR backscatter on Kennicott Glacier from 2017-2024. Select scenes are shown from April through October 2020. (b, c) Automated delineation of melt extents and snowlines shown atop the heatmap plots of all scenes on Kennicott Glacier for path 14, frame 387 using (b) equal-elevation and (c) equal-area bins. Melt extents and snowlines are also shown on spatially distributed maps (a), generally appearing above and below the dark red areas, respectively, with thin lines representing the minimum and maximum values, per the uncertainty.

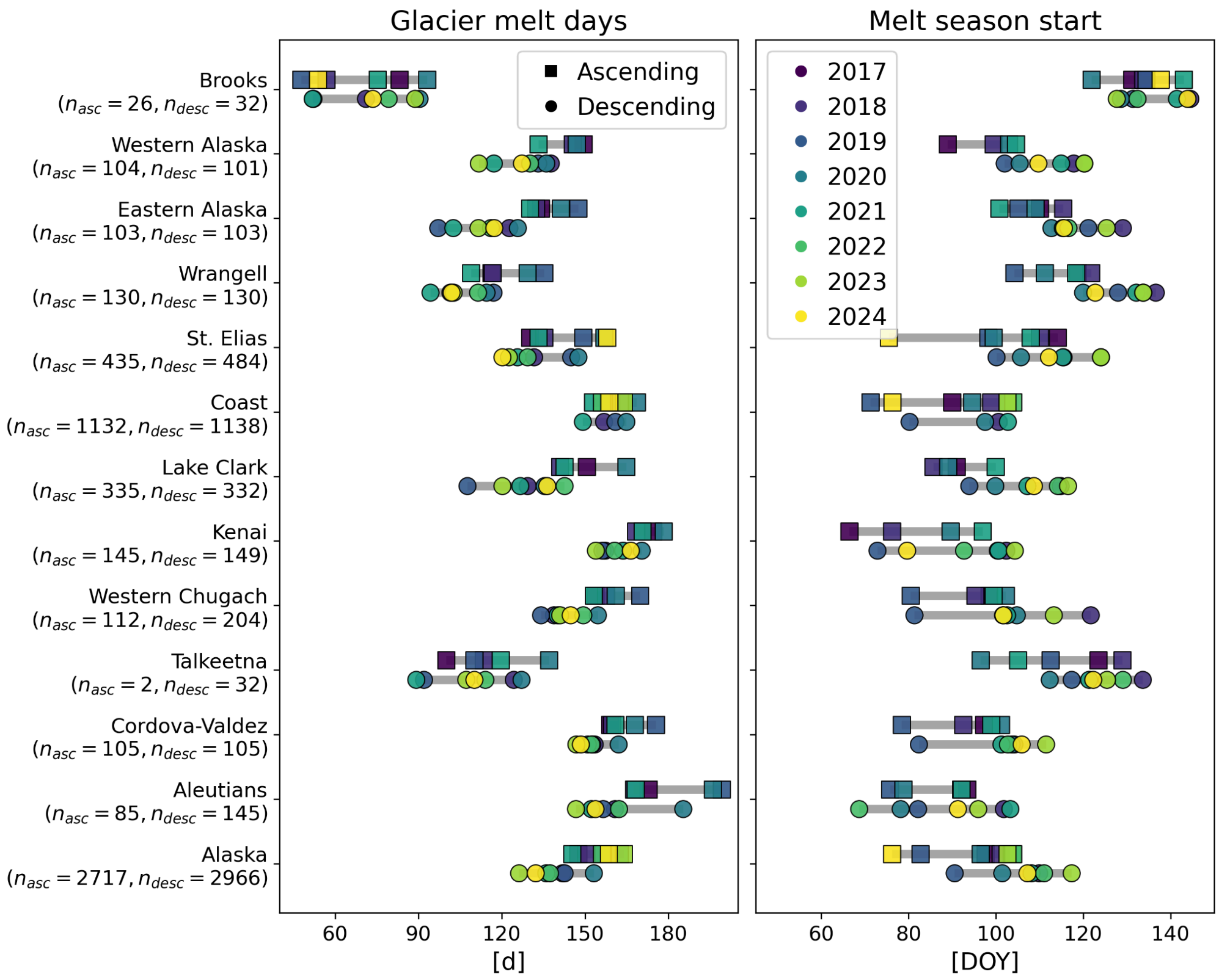

Fig. S2: Glacier melt days and melt season start (date when melt extent exceeds median elevation) for ascending and descending scenes across all subregions of Alaska from 2017-2024.

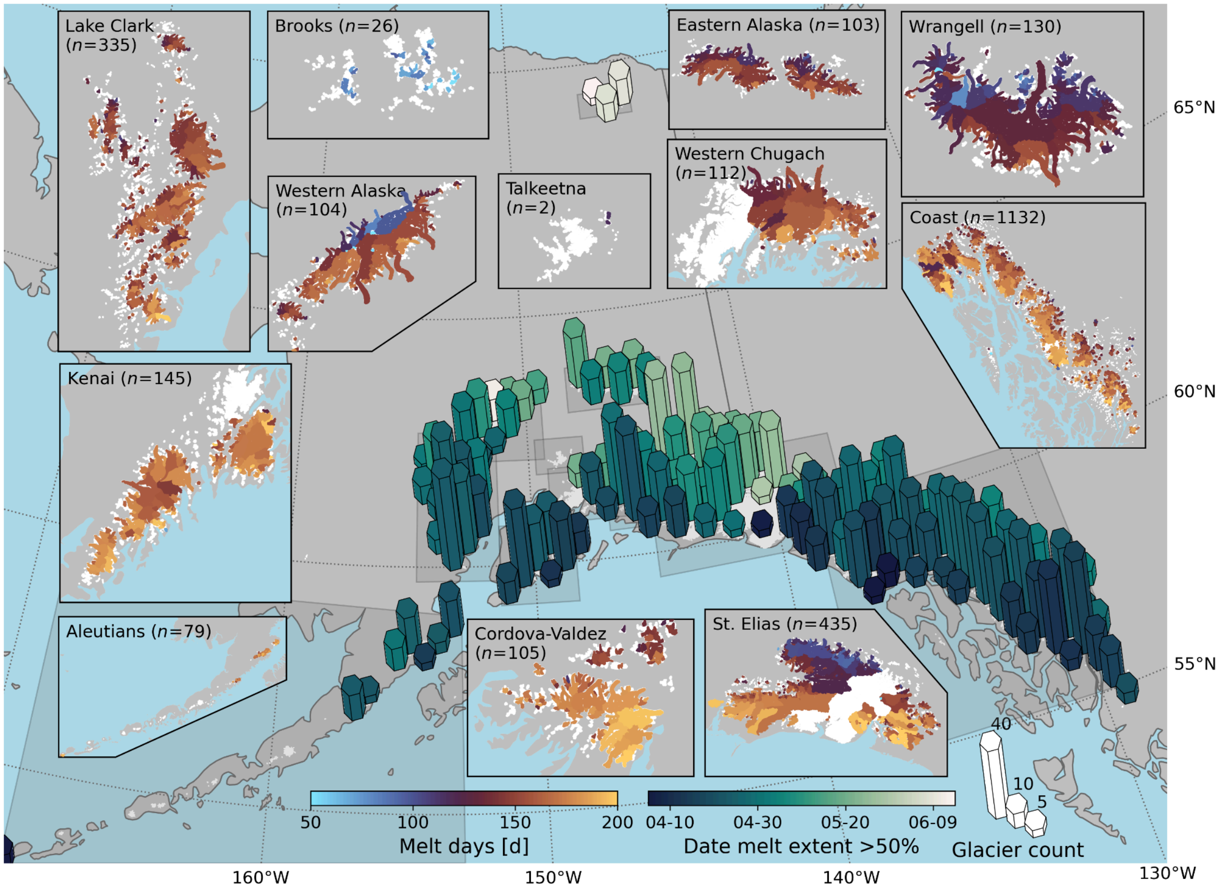

Fig. S3: Glacier melt days and melt season start (date when melt extent exceeds median elevation) for ascending and descending scenes across all subregions of Alaska from 2017-2024.

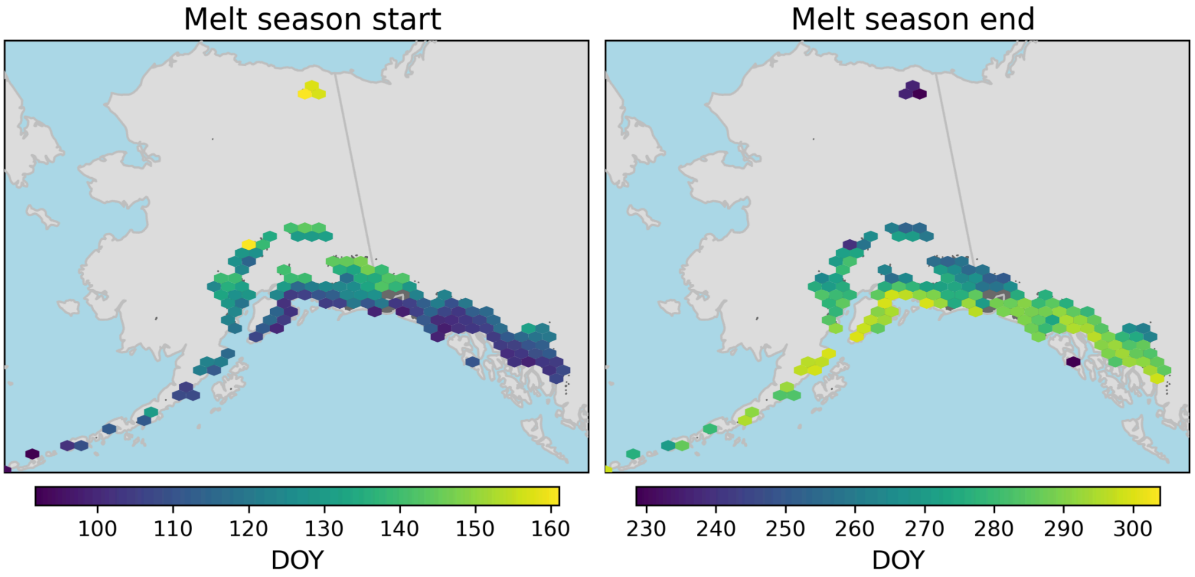

Fig. S4: Melt season start and end, as determined by the first and last occurrence of the melt extent exceeding 50% of the glacier elevation range for descending scenes, thus approximately representing melt in the accumulation area.

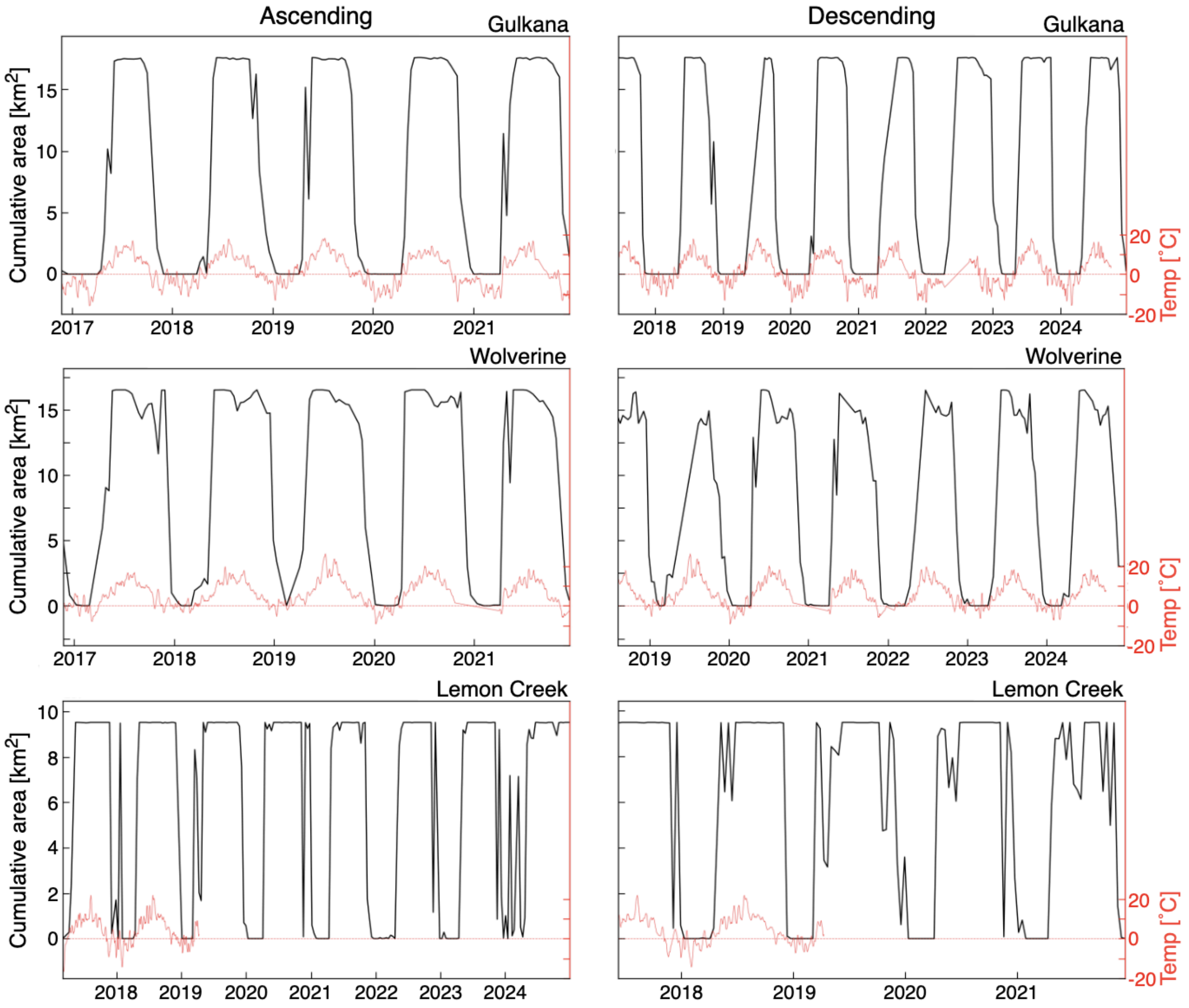

Fig. S5: Glacier melt extent and automated weather station temperature data[7] for the U.S. Geological Survey benchmark glaciers in Alaska for ascending and descending scenes.

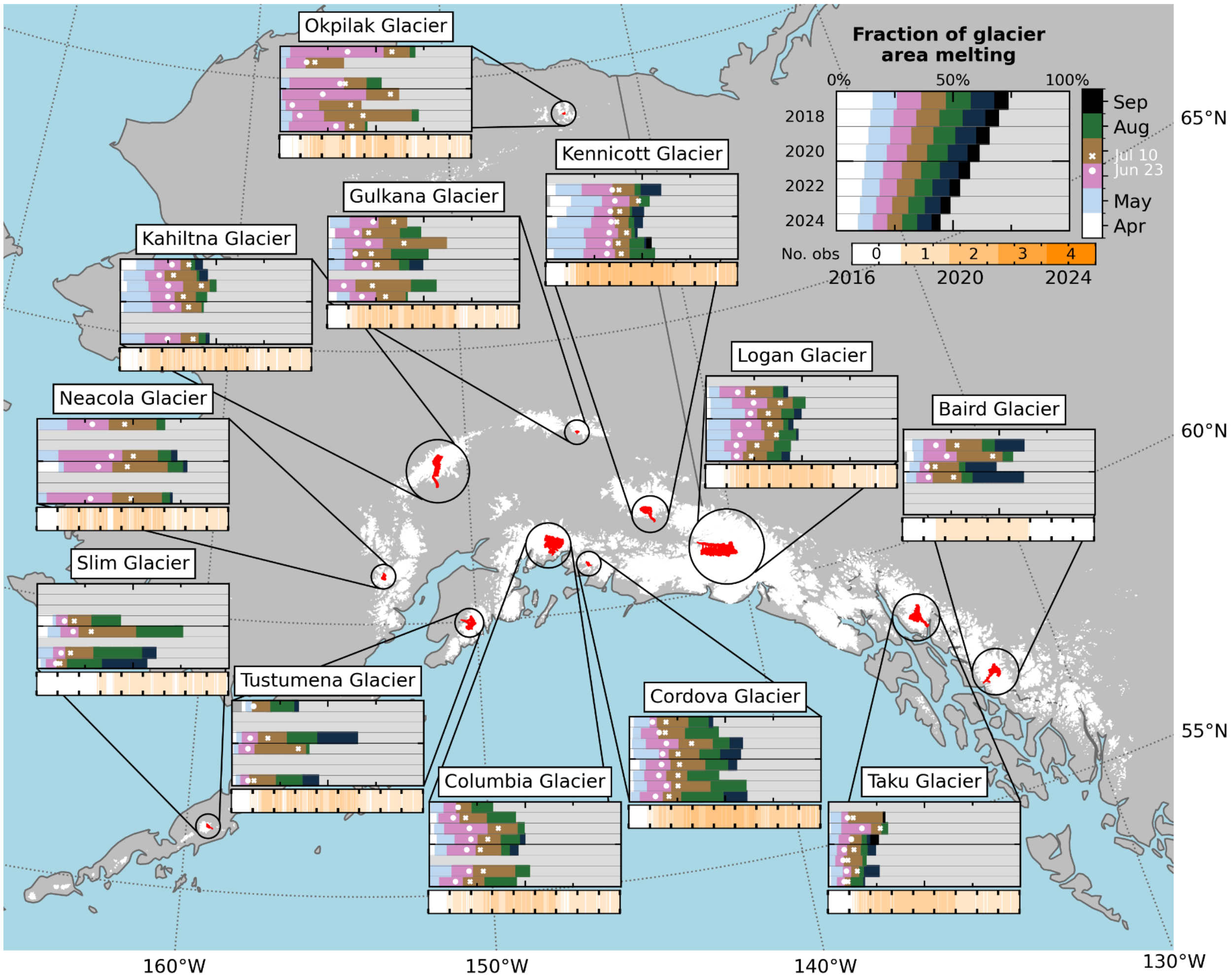

Fig. S6: Transient snowline area fraction for select glaciers across Alaska from 2017-2024. Color indicates the timing of snowline retreat. The bar beneath ring plots shows the number of SAR observations for the glacier over time.

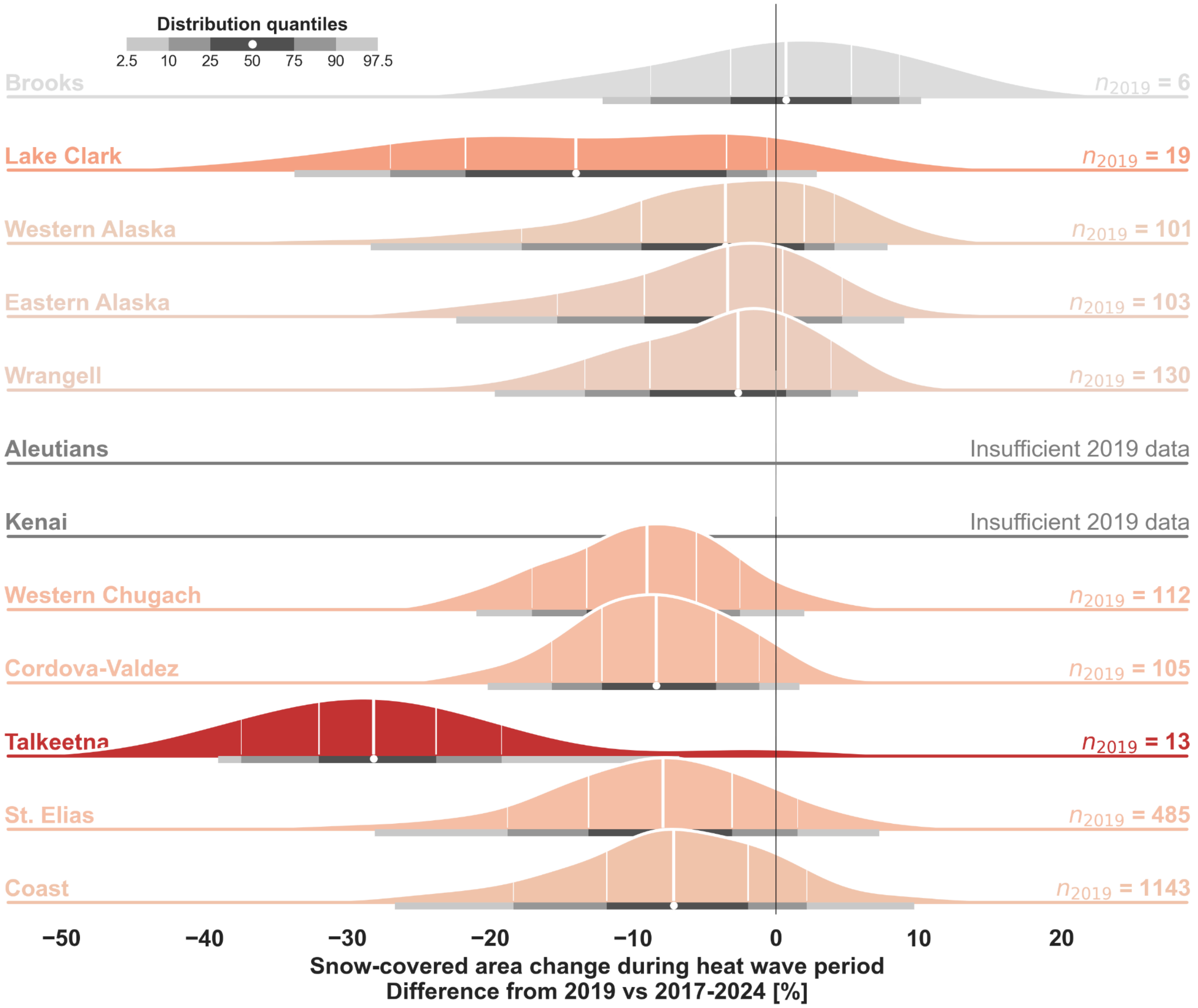

Fig. S7: Snow-covered area change during the heat wave period (June 23 - July 10) in 2019 compared to all other years 2017-2024. Distributions represent values for individual glaciers within each subregion of Alaska.

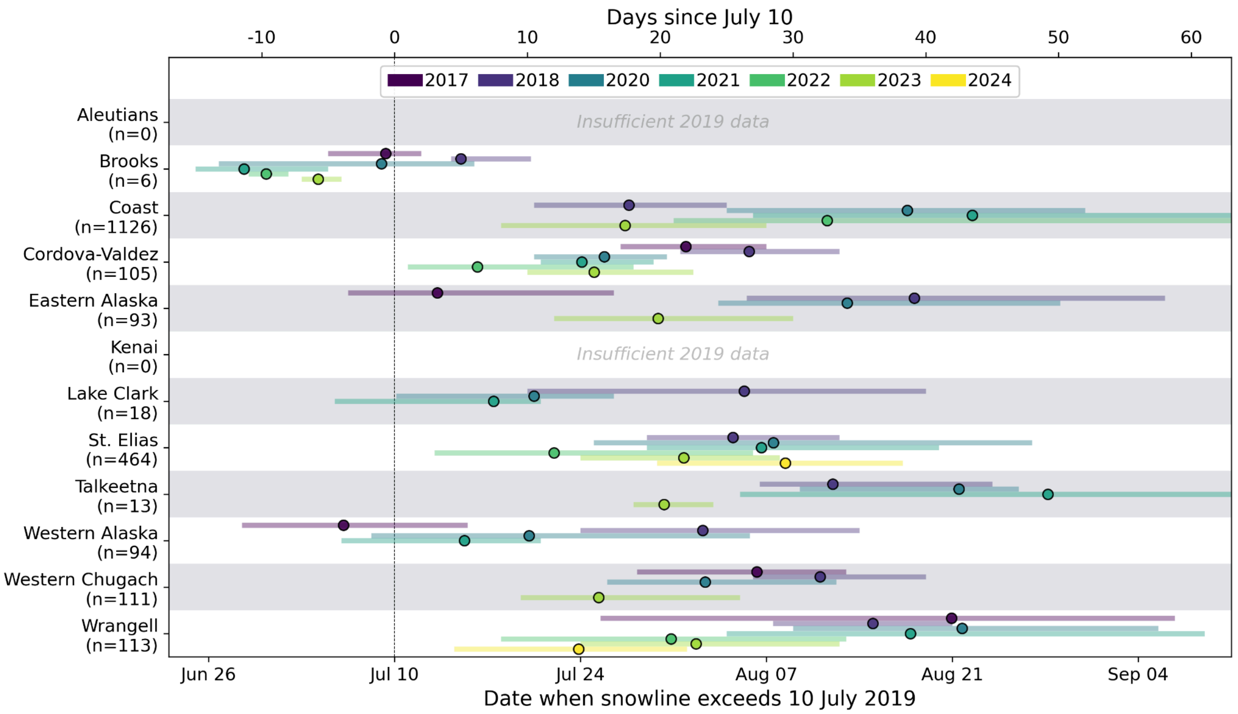

Fig. S8: Difference between 2019 end of heat wave (July 10) snowline and the date when the July 10, 2019 snowline was reached in other years. The bars span the interquartile range of the data.

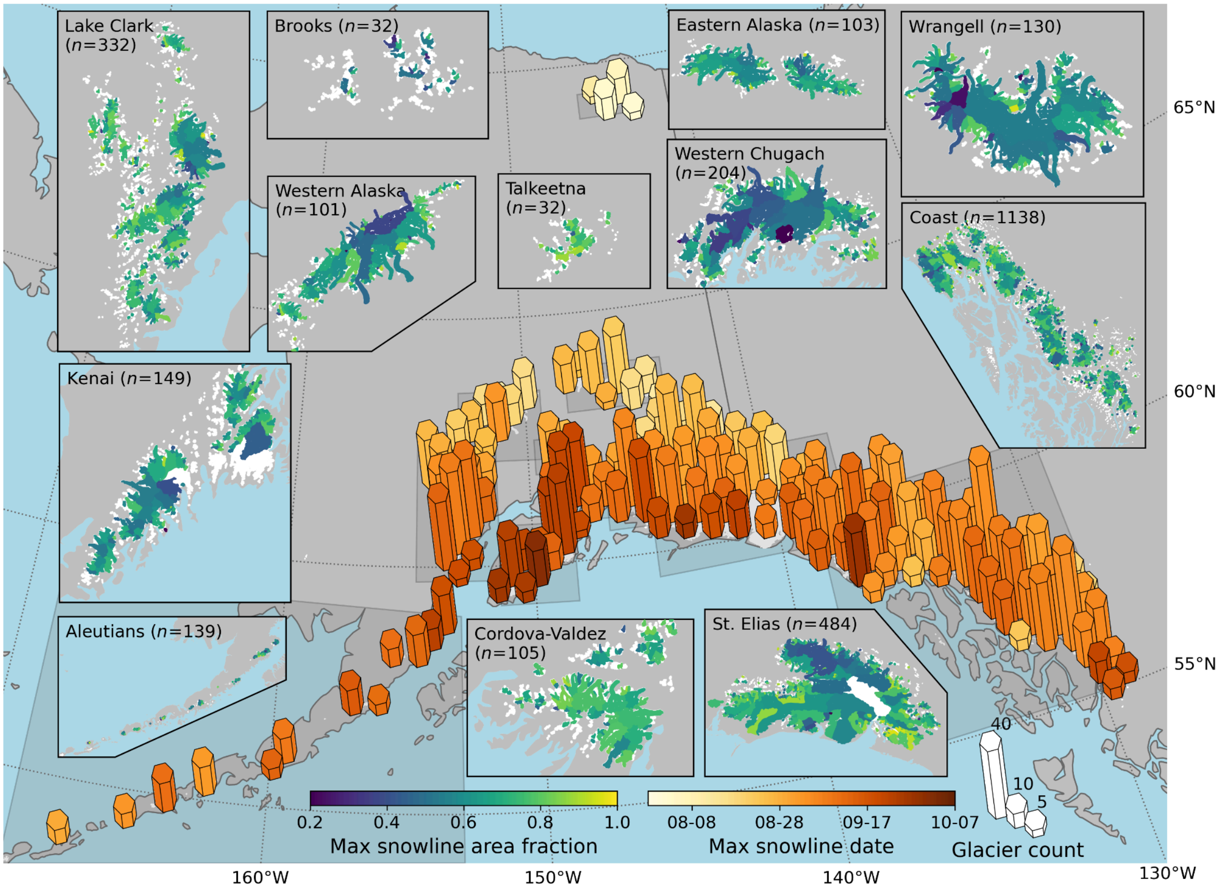

Fig. S9: Maximum snowline date and area fraction across Alaska. Bar height indicated the number of glaciers in each bin. Data represents the 2017-2024 mean values from descending scenes only.

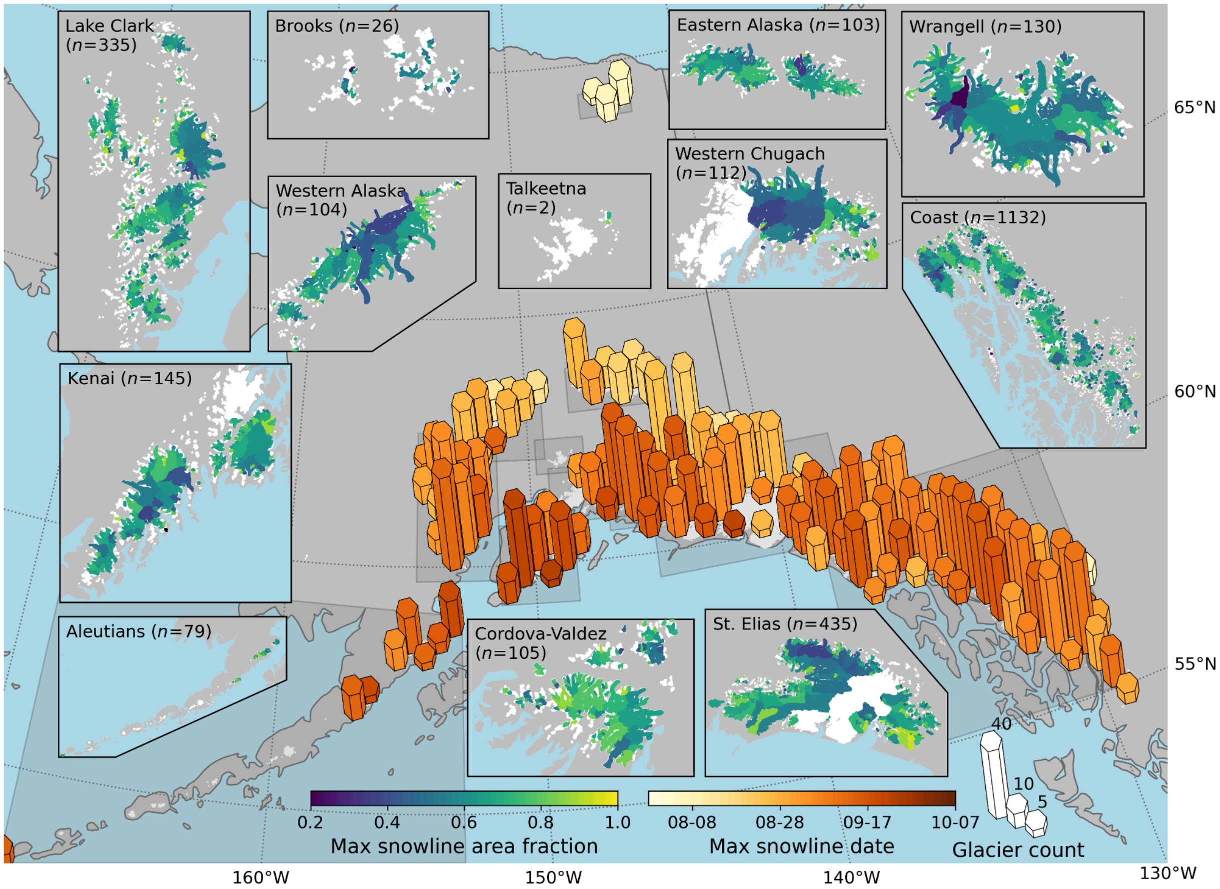

Fig. S10: Maximum snowline date and area fraction across Alaska. Bar height indicated the number of glaciers in each bin. Data represents the 2017-2024 mean values from ascending scenes only.

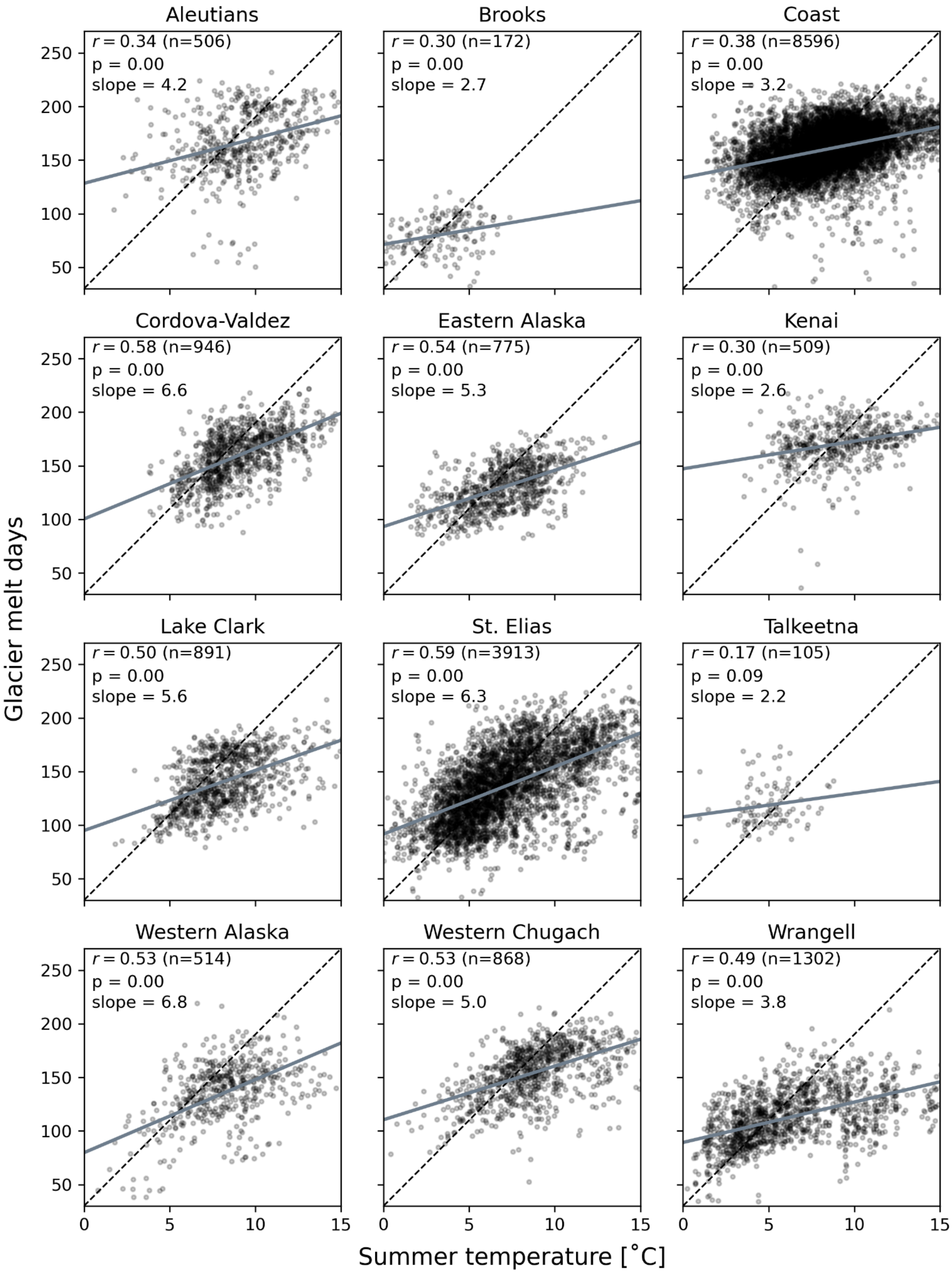

Fig. S11: Correlation between summer temperature (May through September) and glacier melt days for each subregion in Alaska. Each observation (n) represents a value obtained for a single glacier in a given year and SAR satellite path.

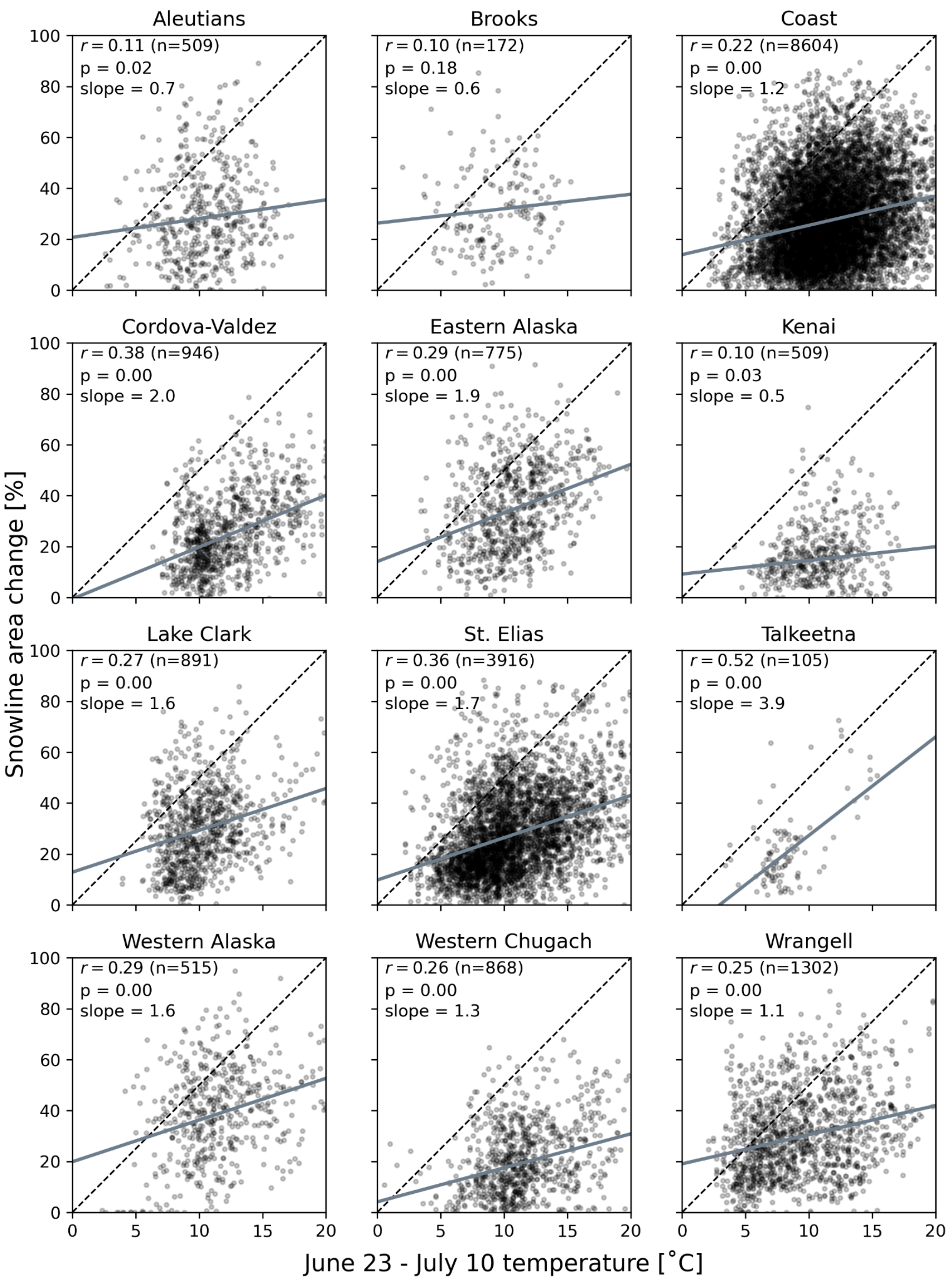

Fig. S12: Correlation between the 2019 heat wave period temperature (June 23 - July 10) and snowline area change up to the end of the heat wave (July 10) for each subregion in Alaska. Each observation (n) represents a value obtained for a single glacier in a given year and SAR satellite path.

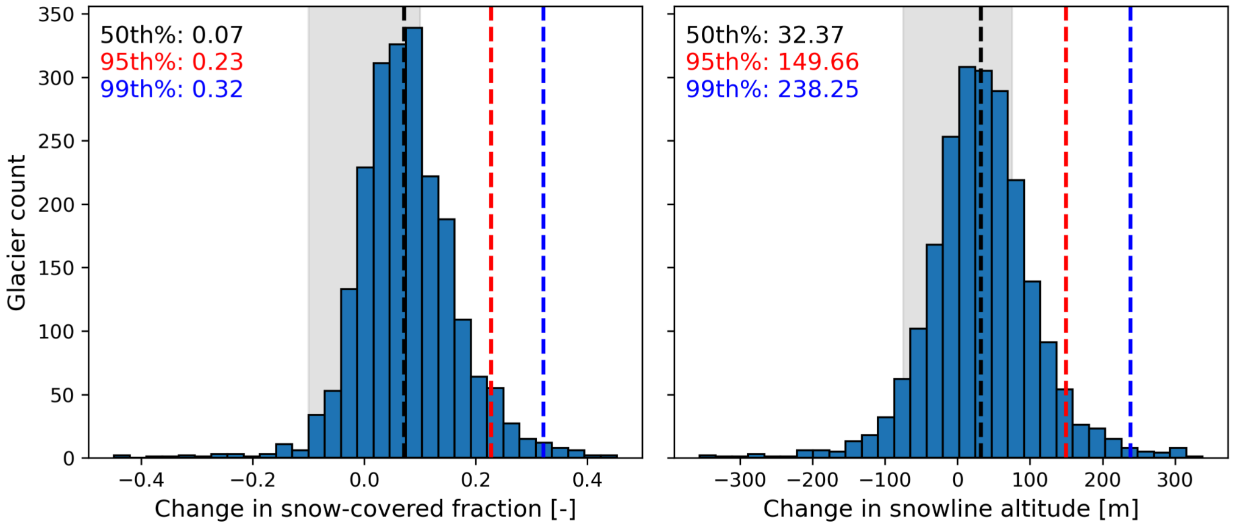

Fig. S13: Difference in snowline change during the heat wave time period for 2019 compared to the mean of other years with available data for a given glacier. Data are shown as a difference in snowline area fraction (left) and elevation (right). The grey area represents the middle quartile of the total range for both metrics.

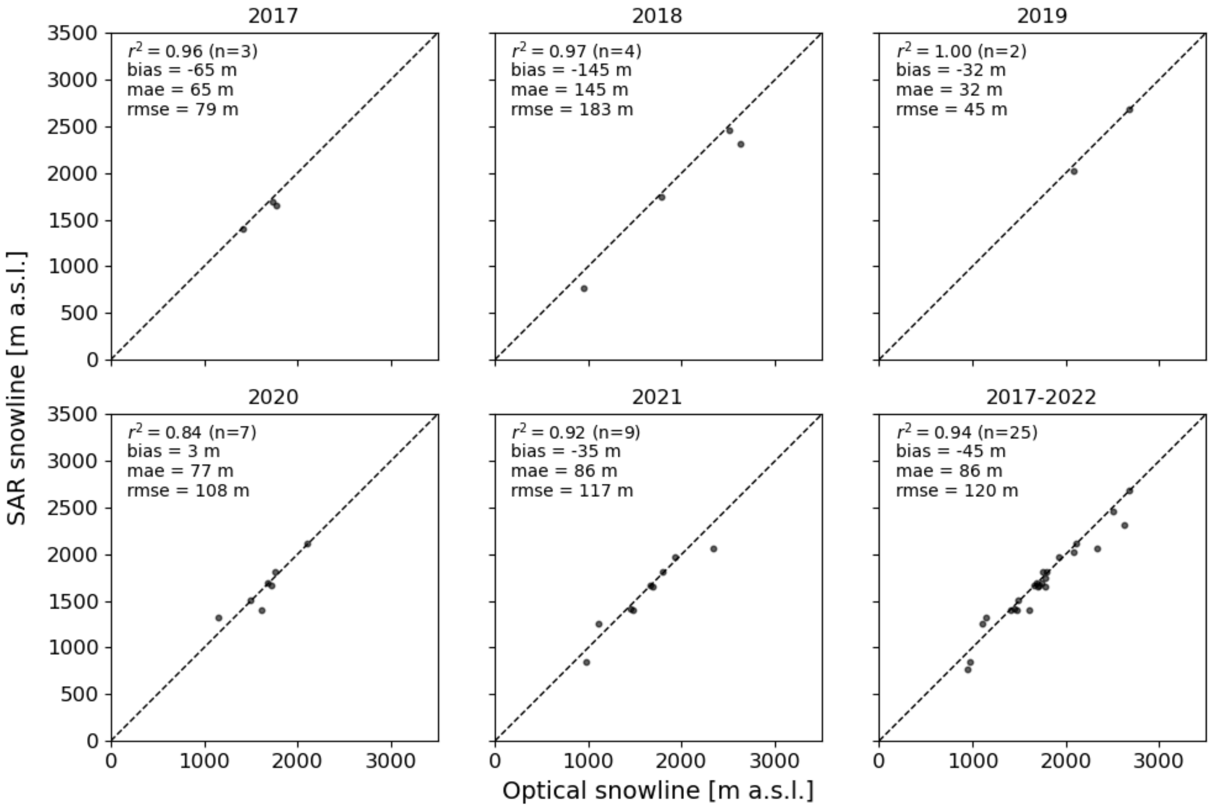

Fig. S14: Comparison between snowlines produced from this study and observations from Larocca et al. (2024)[1], from 2017-2022.

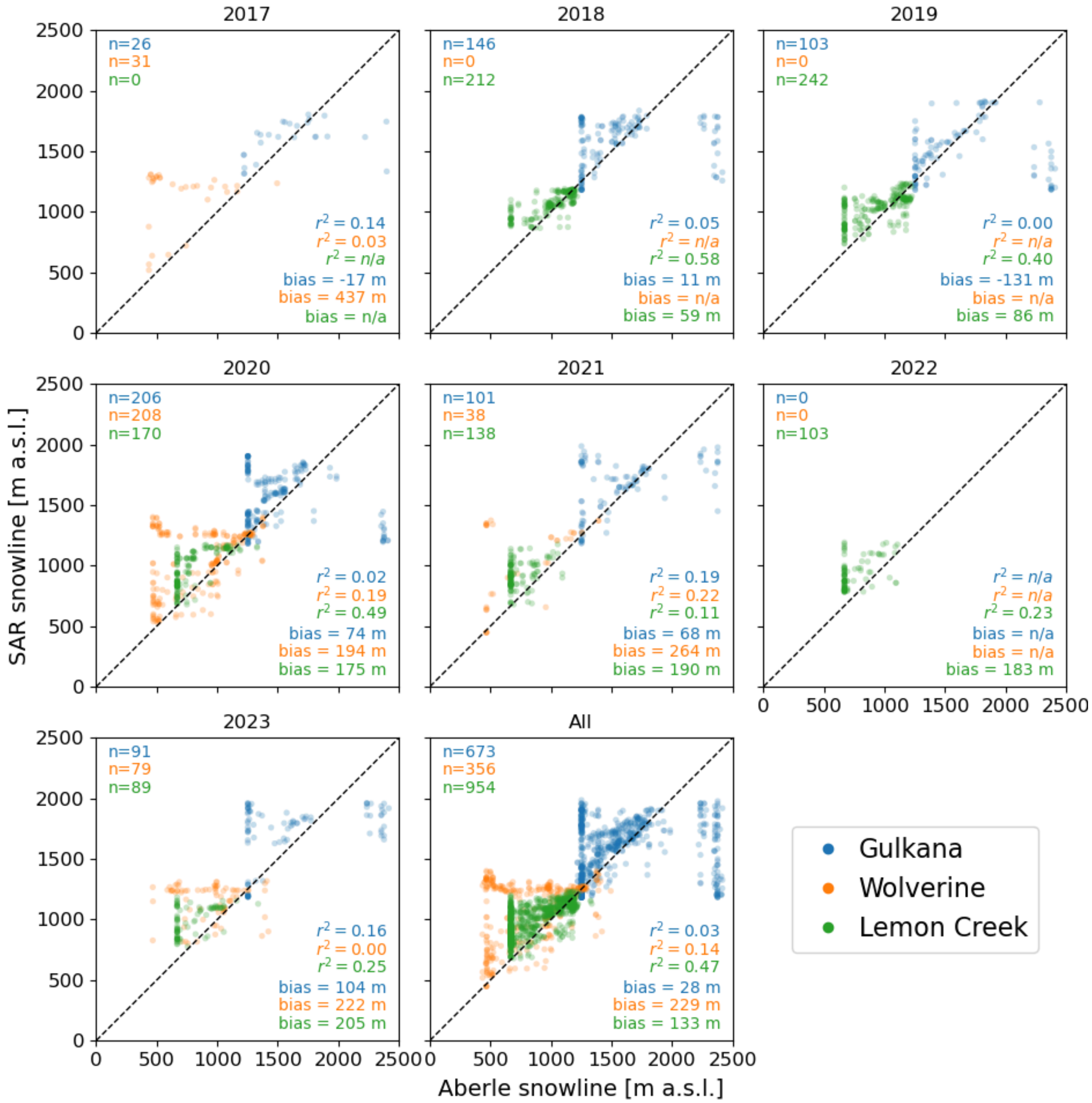

Fig. S15: Comparison between snowlines produced from this study and observations from Aberle et al. (2025a)[3], from 2017-2023.

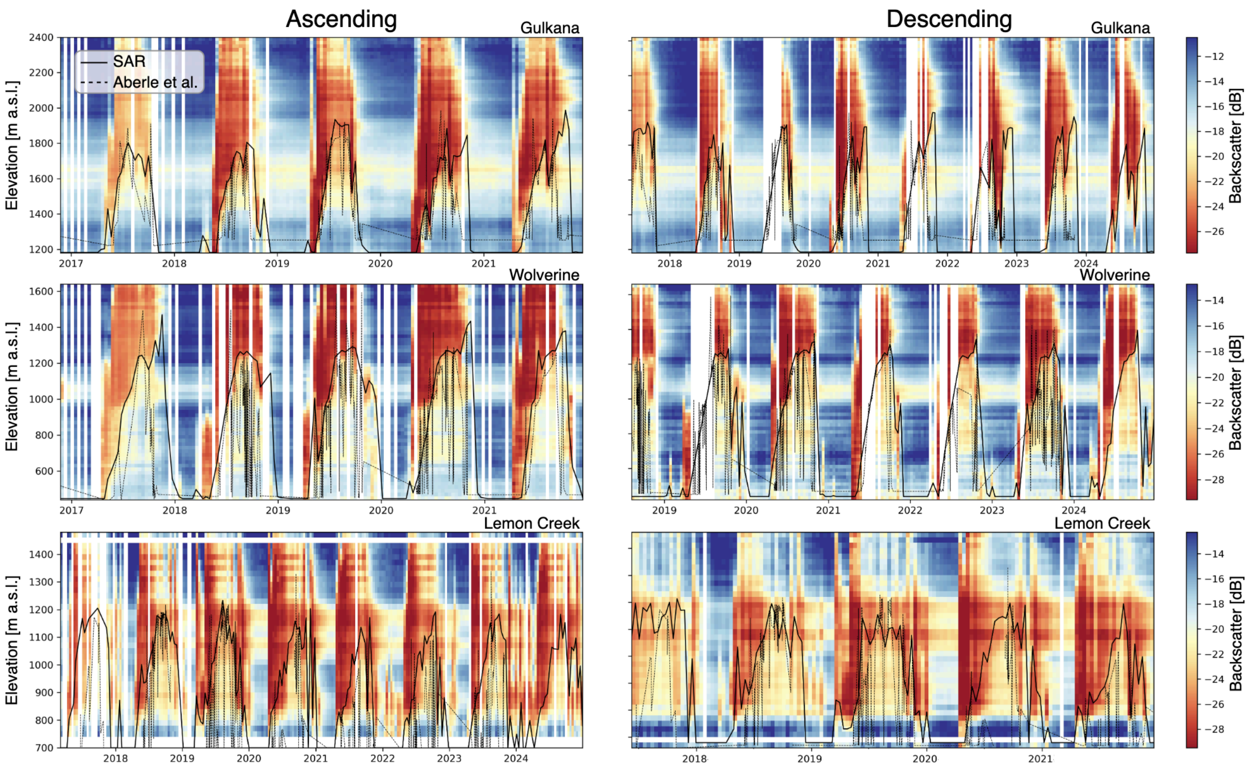

Fig. S16: Transient snowline plots for Gulkana, Wolverine, and Lemon Creek glaciers for this study and observations from Aberle et al. (2025a)[3].

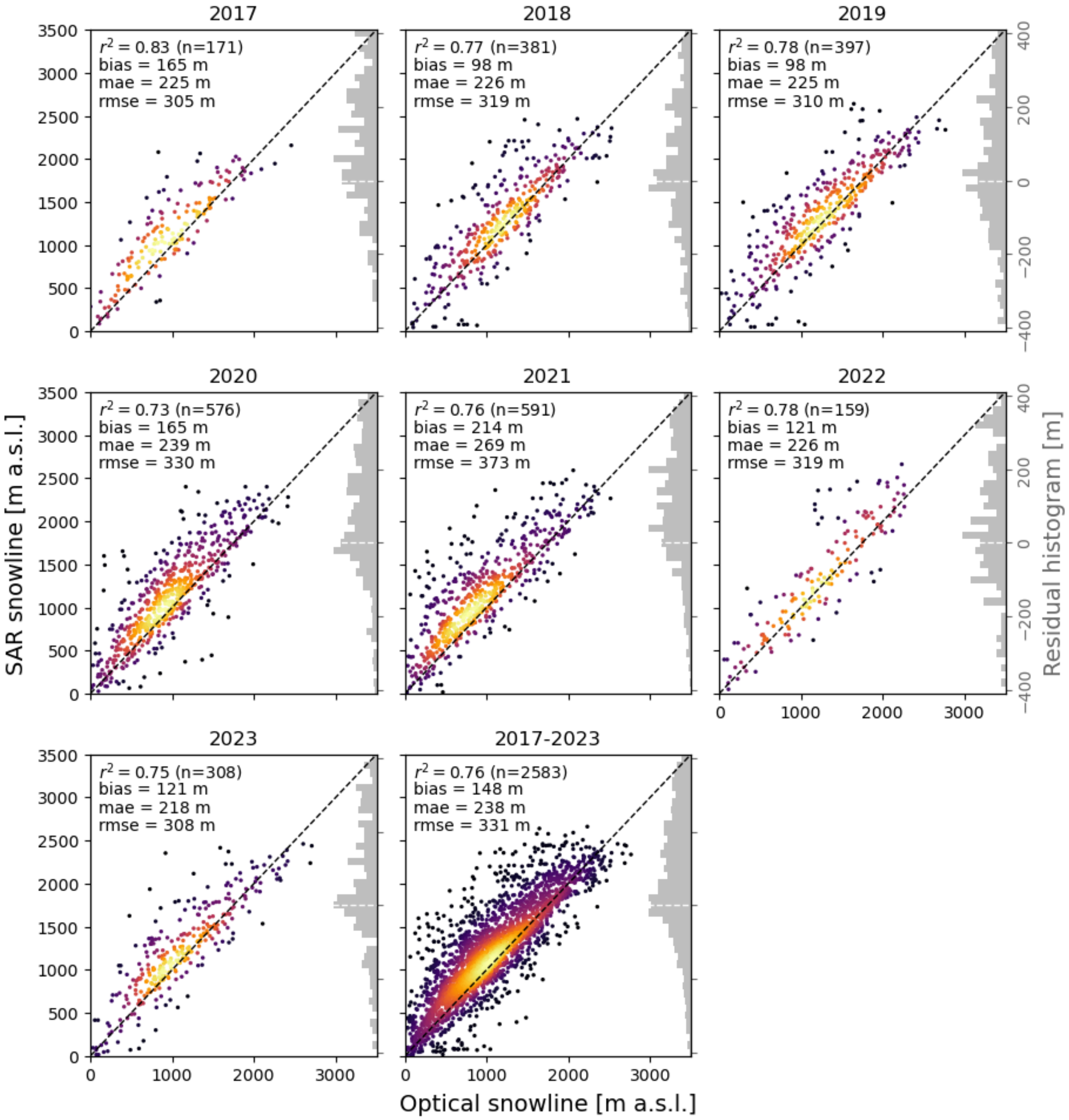

Fig. S17: Comparison between snowlines produced from this study and observations from Aberle et al. (2025b)[4], from 2017-2023. Color represents scatterplot density, with warmer colors indicating a greater number of observations.

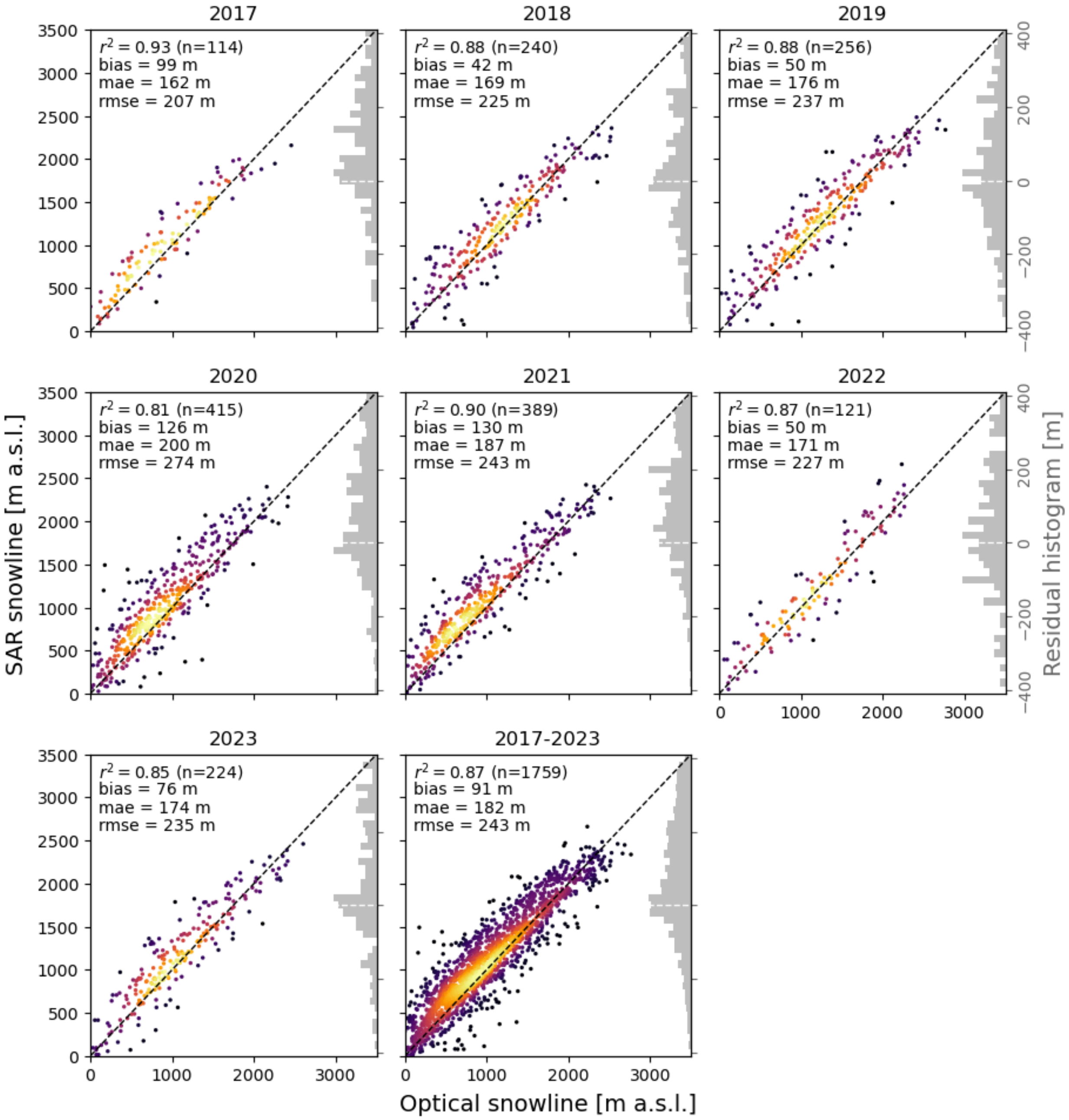

Fig. S18: Comparison between snowlines produced from this study and observations from Aberle et al. (2025b)[4], from 2017-2023. Only snowline observations prior to September are considered. Color represents scatterplot density, with warmer colors indicating a greater number of observations.

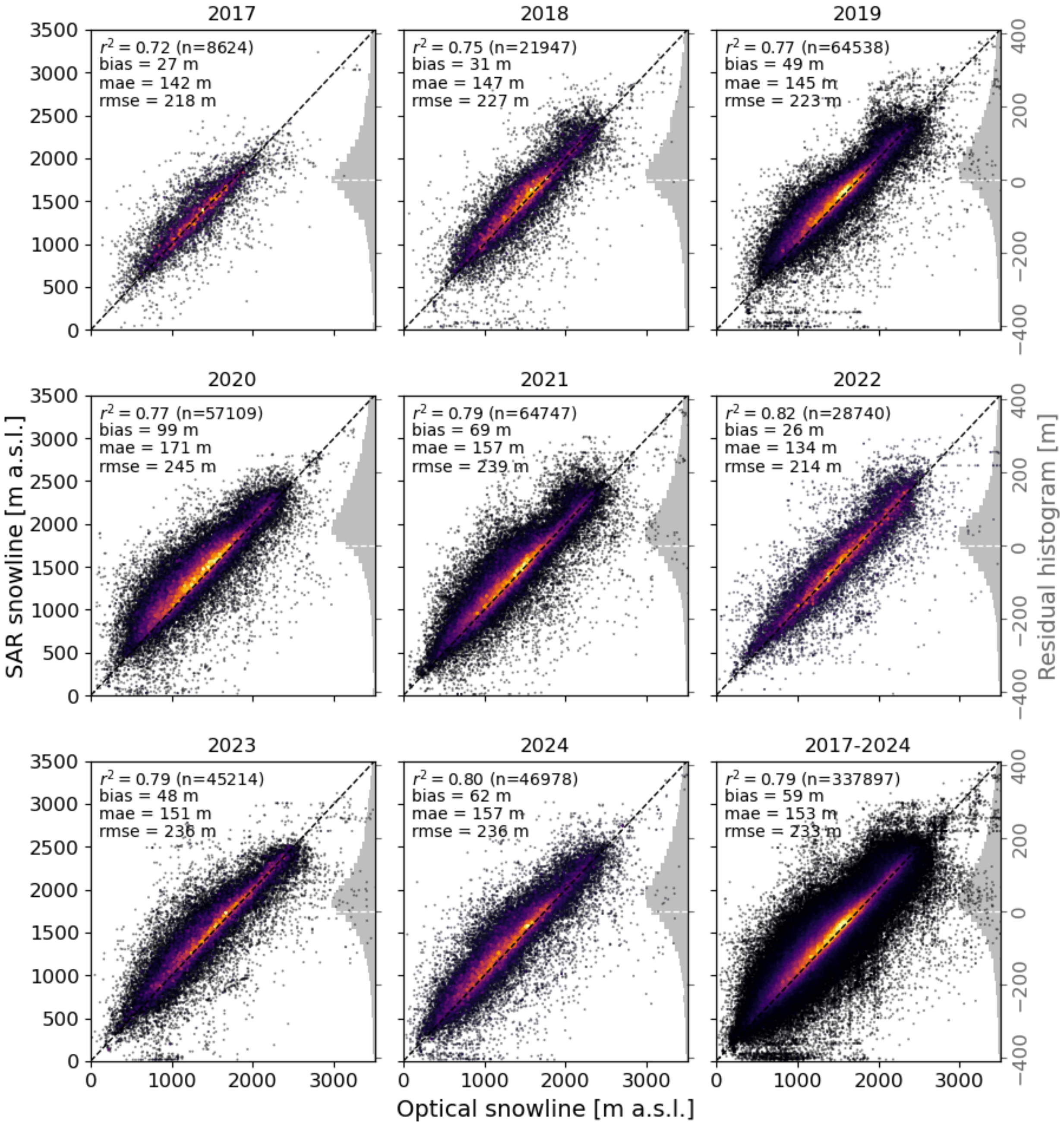

Fig. S19: Comparison between snowlines produced from this study and observations from Bevington and Menounos (2025)[5], from 2017-2024. Color represents scatterplot density, with warmer colors indicating a greater number of observations.

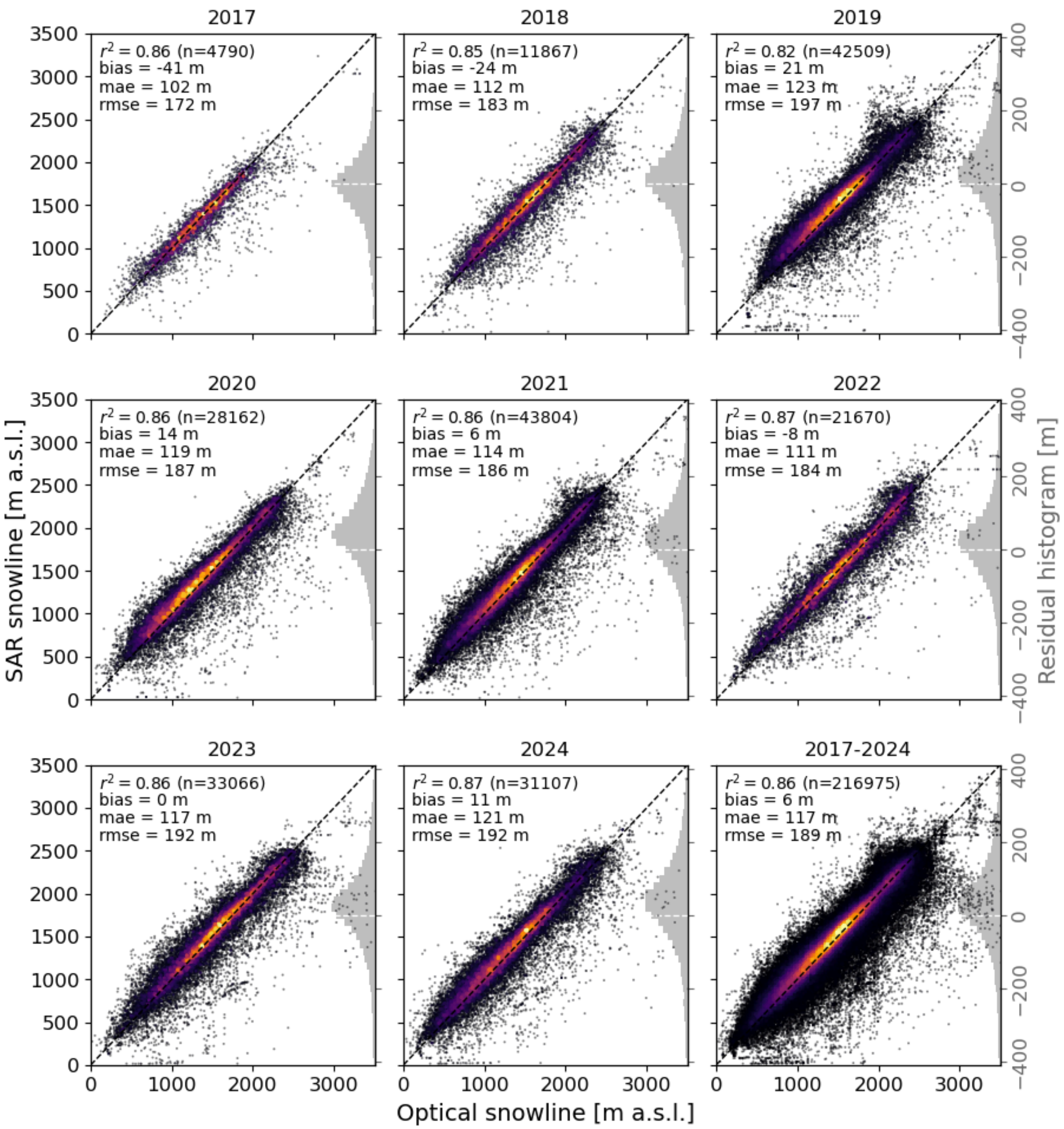

Fig. S20: Comparison between snowlines produced from this study and observations from Bevington and Menounos (2025)[5], from 2017-2024. Only snowline observations from June, July, and August are considered. Color represents scatterplot density, with warmer colors indicating a greater number of observations.

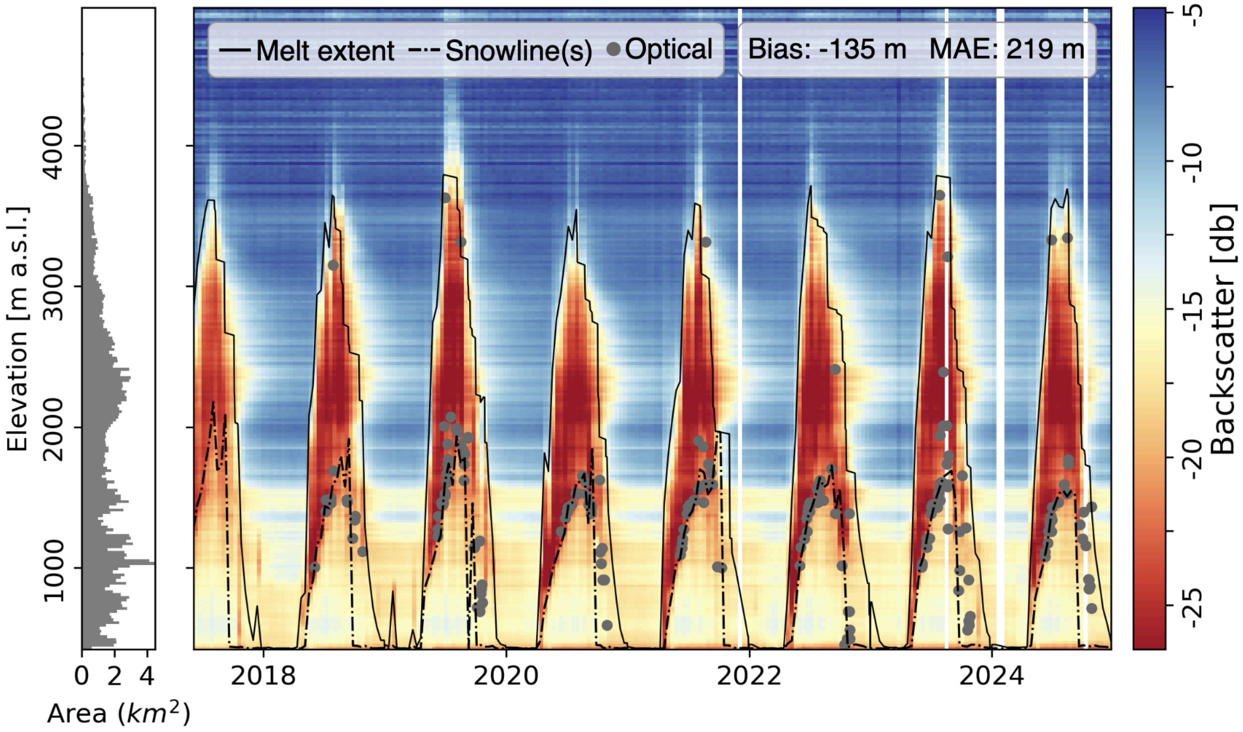

Fig. S21: Sample comparison between snowlines produced from this study and optical sources (ref. 5) on Kennicott Glacier, Alaska. Glacier hypsometry is shown on the left.

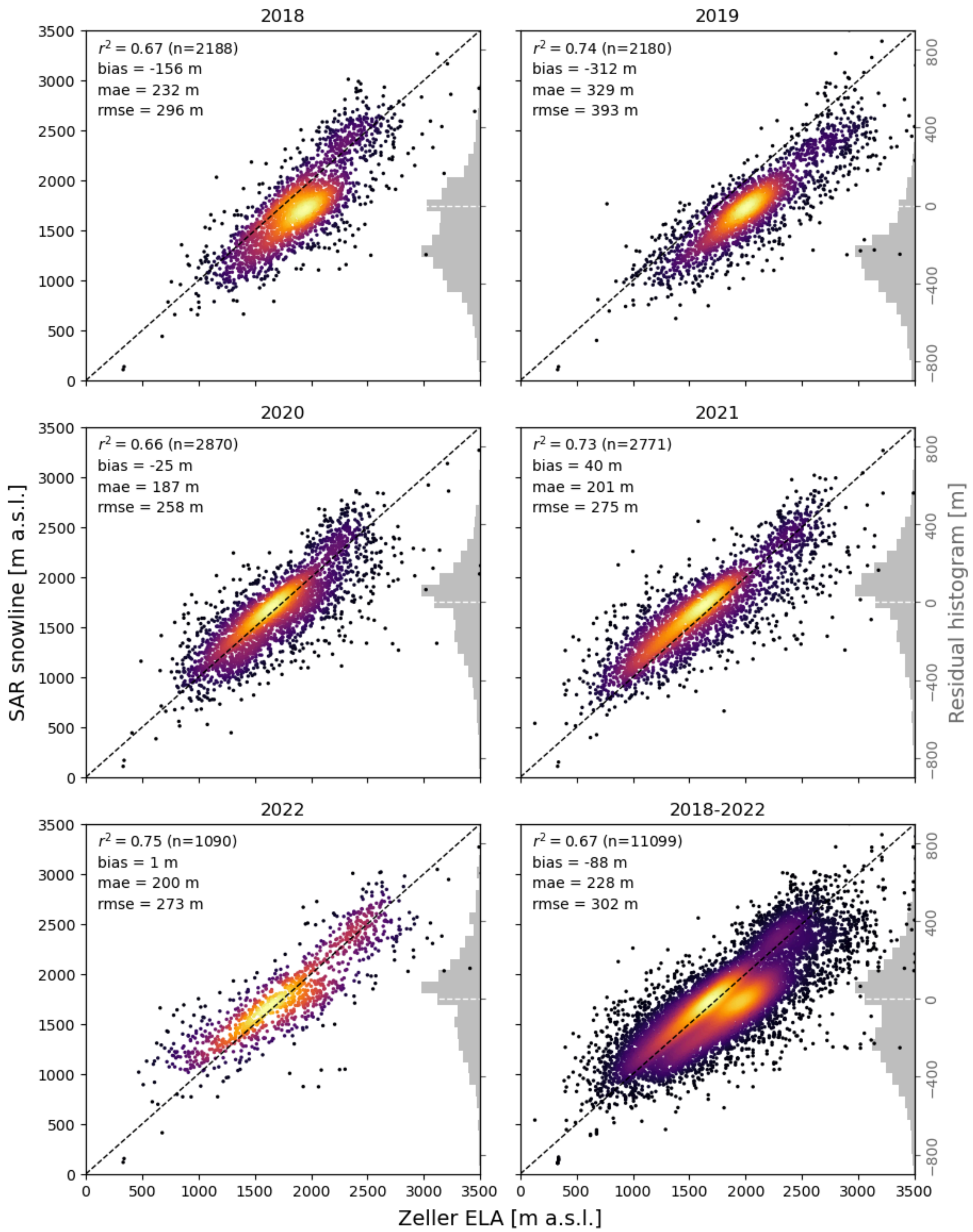

Fig. S22: Comparison between maximum annual snowline produced from this study and equilibrium-line altitude observations from Zeller et al. (2025)[2], from 2018-2022. Color represents scatterplot density, with warmer colors indicating a greater number of observations.

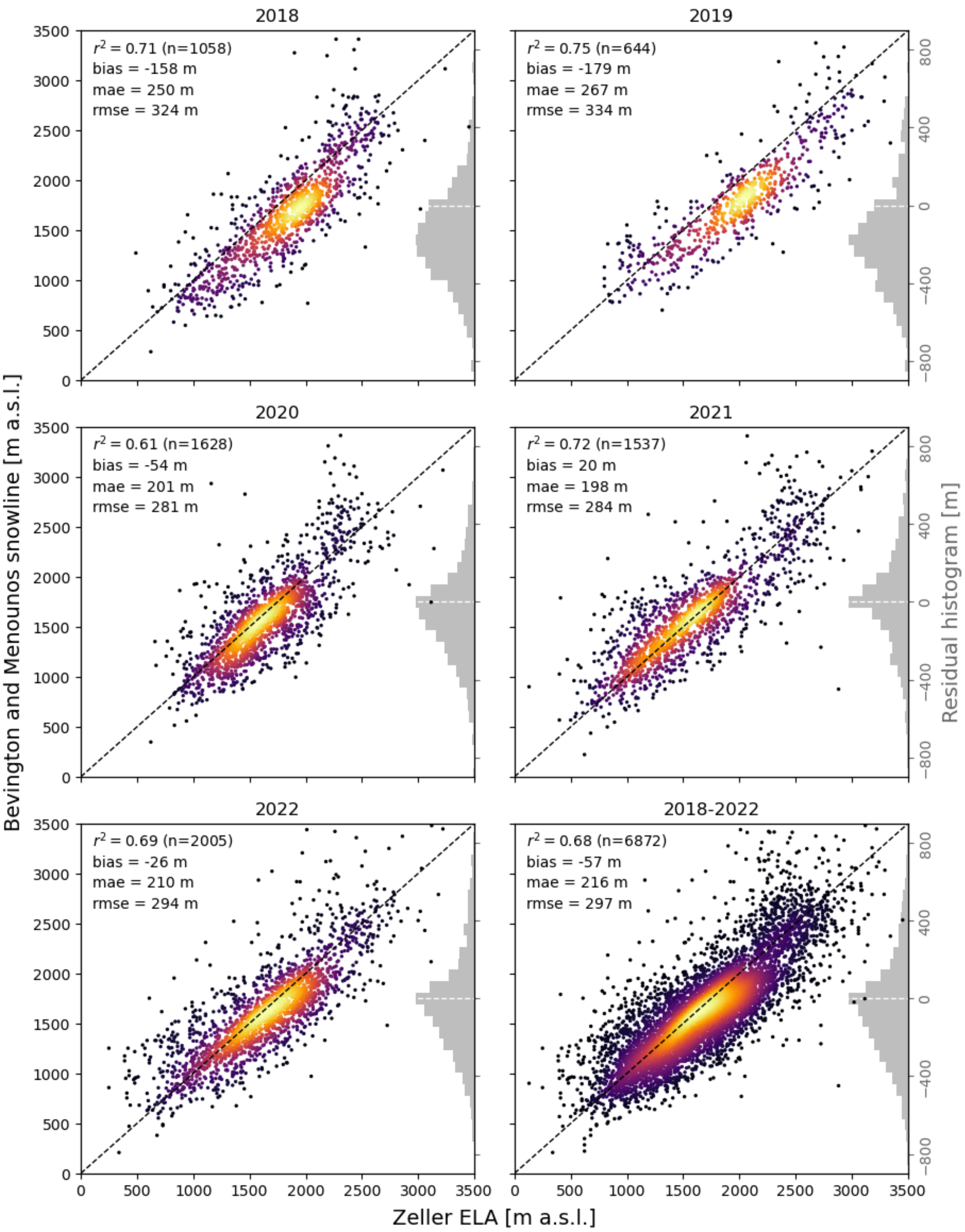

Fig. S23: Comparison between maximum annual snowline from Bevington and Menounos (2025)[5] and equilibrium-line altitude observations from Zeller et al. (2025)[2], from 2018-2022. Color represents scatterplot density, with warmer colors indicating a greater number of observations.

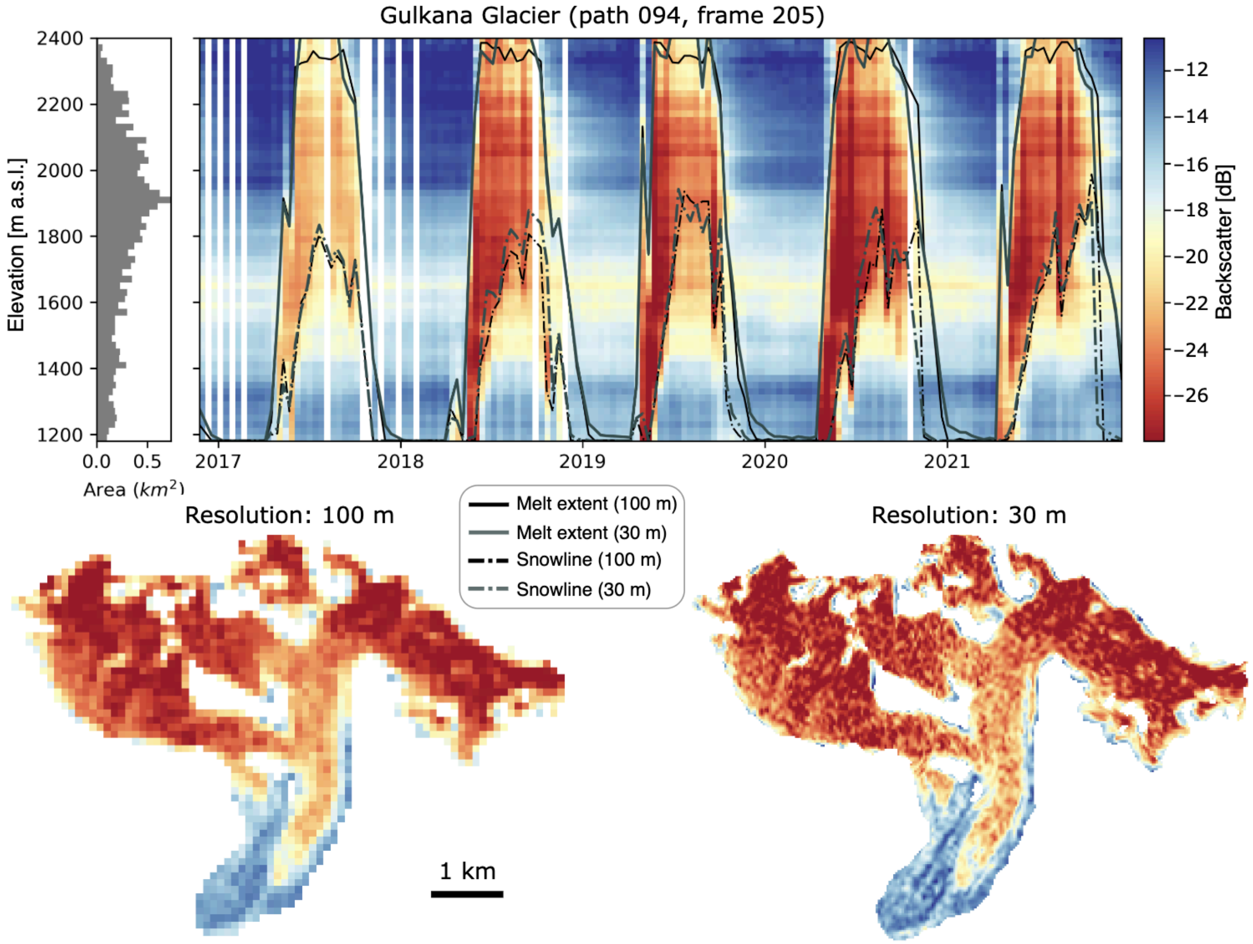

Fig. S24: Example of Sentinel-1 SAR backscatter images and automatically-detected melt extents and snowline altitudes for Gulkana Glacier at native 30 m resolution and coarsened 100 m resolution. Spatially-distributed backscatter images are shown for August 10, 2018.

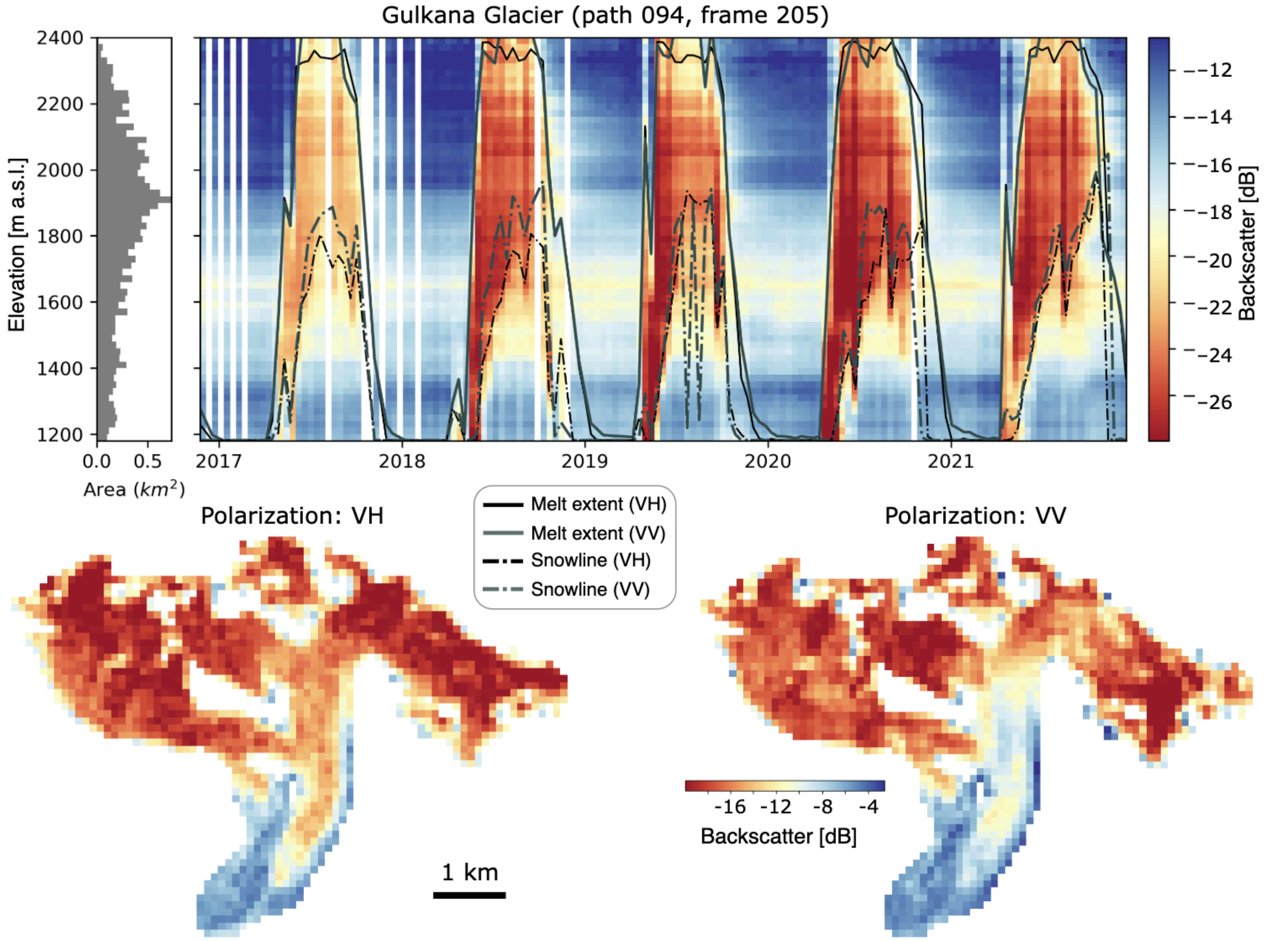

Fig. S25: Example of Sentinel-1 SAR backscatter images and automatically-detected melt extents and snowline altitudes for Gulkana Glacier with cross-polarized (VH) and co-polarized (VV) SAR. Spatially-distributed backscatter images are shown for August 10, 2018. Note that the range of backscatter values for the co-polarized image differs from the cross-polarized SAR.

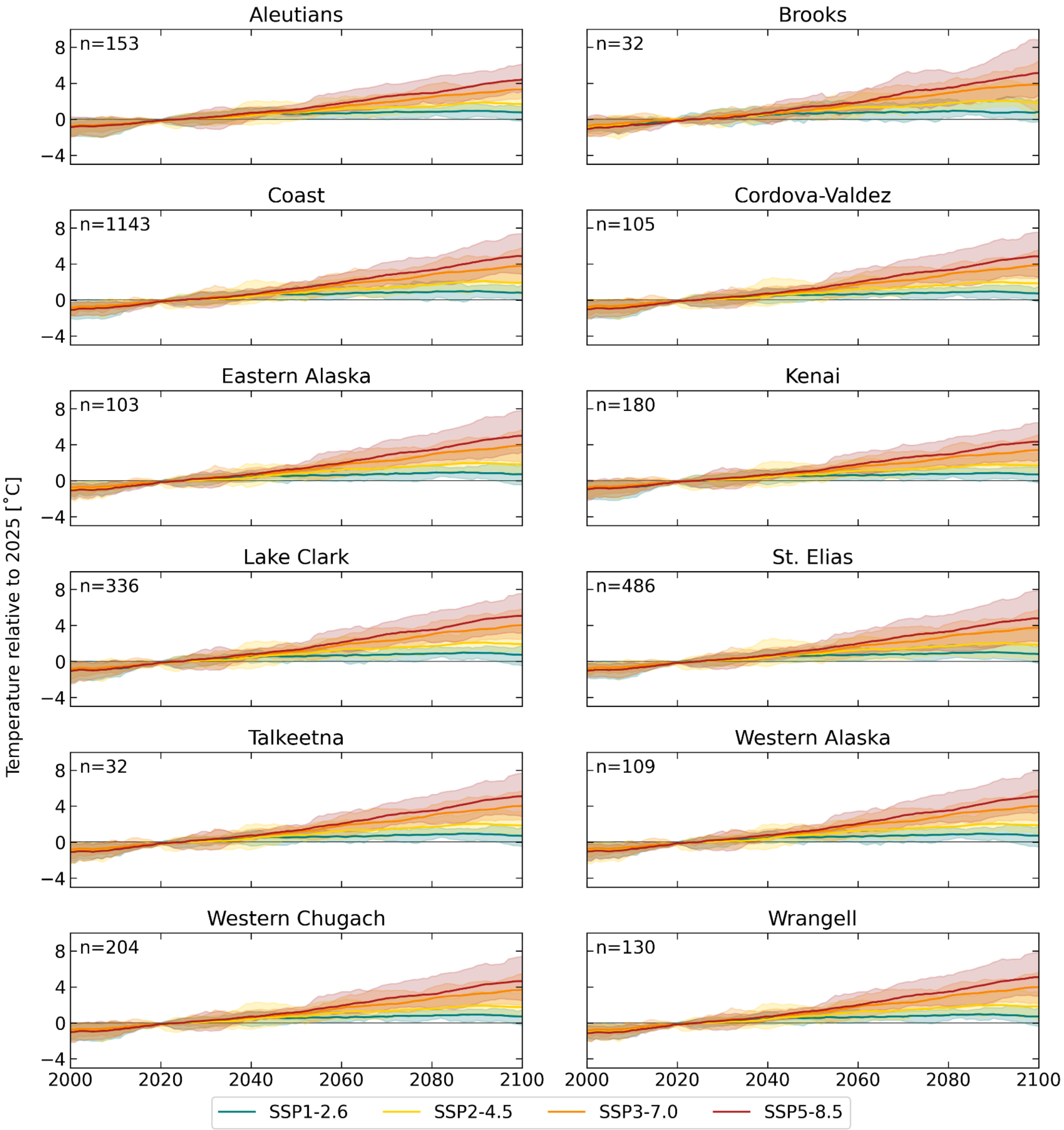

Fig. S26: Projected mean summer (May through September) warming from 2000-2100 for each subregion of Alaska, relative to 2025. Temperatures are from an ensemble of 12 GCMs and four SSPs for all glaciers with processed SAR data in each subregion. The shaded region represents the mean minimum and maximum value from the GCMs.

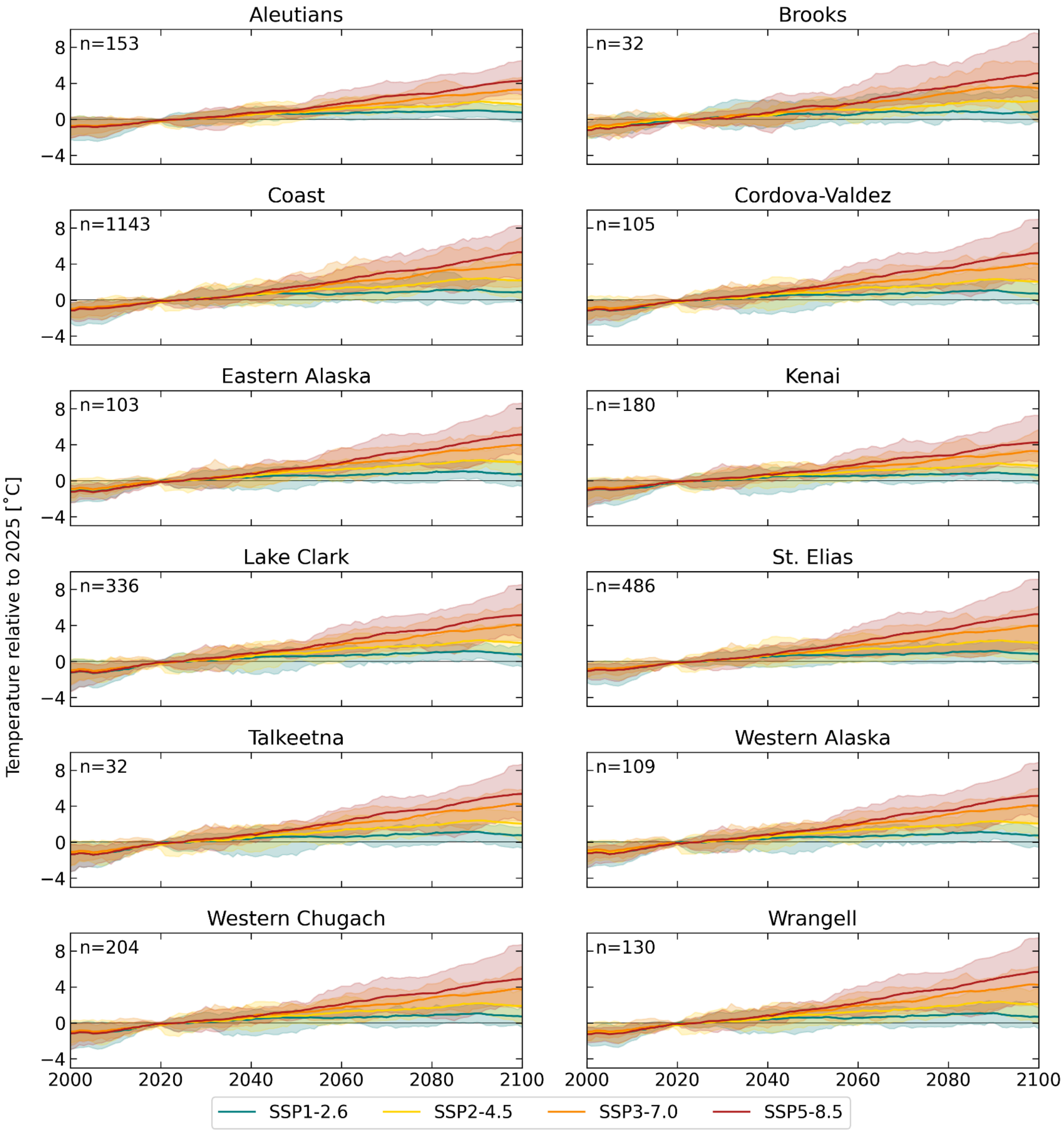

Fig. S27: Projected mean June and July warming from 2000-2100 for each subregion of Alaska, relative to 2025. Temperatures are from an ensemble of 12 GCMs and four SSPs for all glaciers with processed SAR data in each subregion. The shaded region represents the mean minimum and maximum value from the GCMs.

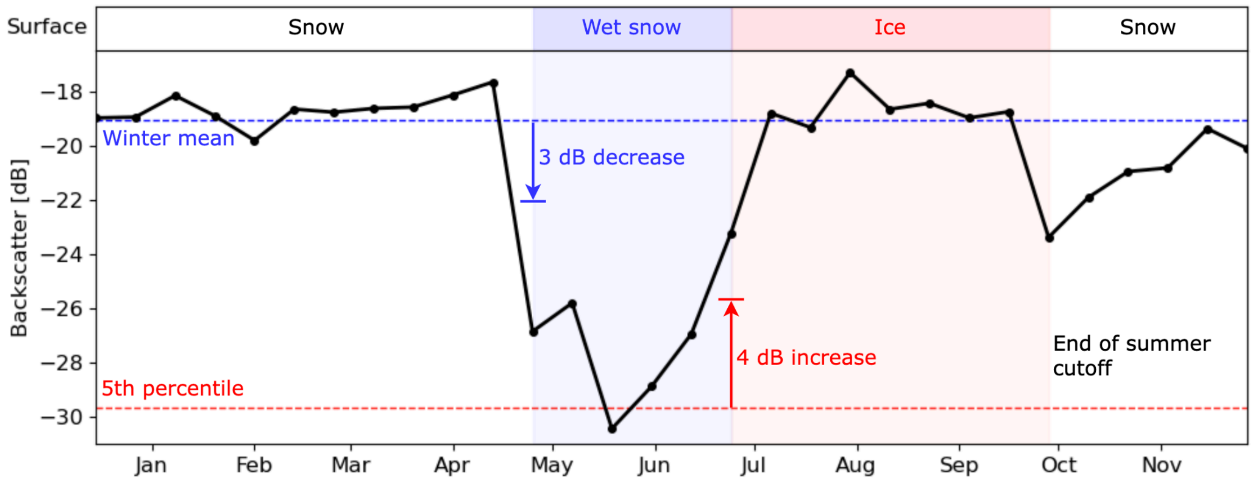

Fig. S28: Sample backscatter for a glaciated pixel over the course of a year. Snow, wet snow, and ice are identified based on reference backscatter values (winter mean and 5th percentile of values) and offset thresholds (-3 dB to identify melt and 4 dB to identify snow-free). Detection of no melt at the end of the summer is determined from pixels glacier-wide or from applying a temperature-based cutoff. Note that the classification of snow at the end of the summer is based on pixels returning to winter values glacier-wide (i.e., no melt is occurring anywhere) or using sub-freezing temperatures as a threshold, which accounts for SAR backscatter seeing wet snow or firn in the early winter due to slow refreezing processes. This cutoff is used as opposed to a specific threshold since SAR sees through dry snow layers such that the backscatter remains consistent before and after dry snowfall.

4 SUPPLEMENTARY MATERIAL REFERENCES

1. Larocca, L. J. *et al.* Arctic glacier snowline altitudes rise 150 m over the last 4 decades. *The Cryosphere* **18**, 3591–3611 (2024).
2. Zeller, L., McGrath, D., Sass, L., Florentine, C. & Downs, J. Equilibrium line altitudes, accumulation areas and the vulnerability of glaciers in Alaska. *Journal of Glaciology* **71**, e28 (2025).
3. Aberle, R. *et al.* Automated snow cover detection on mountain glaciers using spaceborne imagery and machine learning. *The Cryosphere* **19**, 1675–1693 (2025).
4. Aberle, R. *et al.* Leveraging Weekly Snow Cover Time Series for Improved Glacier Monitoring and Modeling. *Geophysical Research Letters* **52**, e2025GL115523 (2025).
5. Bevington, A. R. & Menounos, B. Glaciers in western North America experience exceptional transient snowline rise over satellite era. *Environ. Res. Lett.* **20**, 054044 (2025).
6. Racoviteanu, A. E., Rittger, K. & Armstrong, R. An Automated Approach for Estimating Snowline Altitudes in the Karakoram and Eastern Himalaya From Remote Sensing. *Front. Earth Sci.* **7**, (2019).
7. U.S. Geological Survey Glacier Project, 2019, High altitude weather station data at USGS Benchmark Glaciers (ver 2.0, December 2024): U.S. Geological Survey data release, https://doi.org/10.5066/P9EUXIPE.
